# *PsEND1* Is a Key Player in Pea Pollen Development Through the Modulation of Redox Homeostasis

**DOI:** 10.3389/fpls.2021.765277

**Published:** 2021-10-29

**Authors:** Rim Hamza, Edelín Roque, Concepción Gómez-Mena, Francisco Madueño, José Pío Beltrán, Luis A. Cañas

**Affiliations:** Department of Plant Development and Hormone Action, Instituto de Biología Molecular y Celular de Plantas (CSIC-Universitat Politècnica de València), Ciudad Politécnica de la Innovación, Valencia, Spain

**Keywords:** *Pisum sativum*, *PsEND1* promoter, anther-specific gene, *cis*-regulatory motifs, CArG boxes, hemopexin-like, redox homeostasis

## Abstract

Redox homeostasis has been linked to proper anther and pollen development. Accordingly, plant cells have developed several Reactive Oxygen Species (ROS)-scavenging mechanisms to maintain the redox balance. Hemopexins constitute one of these mechanisms preventing heme-associated oxidative stress in animals, fungi, and plants. *Pisum sativum ENDOTHECIUM 1 (PsEND1*) is a pea anther-specific gene that encodes a protein containing four hemopexin domains. We report the functional characterization of *PsEND1* and the identification in its promoter region of *cis*-regulatory elements that are essential for the specific expression in anthers. *PsEND1* promoter deletion analysis revealed that a putative CArG-like regulatory motif is necessary to confer promoter activity in developing anthers. Our data suggest that *PsEND1* might be a hemopexin regulated by a MADS-box protein. *PsEND1* gene silencing in pea, and its overexpression in heterologous systems, result in similar defects in the anthers consisting of precocious tapetum degradation and the impairment of pollen development. Such alterations were associated to the production of superoxide anion and altered activity of ROS-scavenging enzymes. Our findings demonstrate that *PsEND1* is essential for pollen development by modulating ROS levels during the differentiation of the anther tissues surrounding the microsporocytes.

## Introduction

Anthers development is a critical step in plant reproduction as it is required for male gametophytes generation and fertility. Anthers are composed of three outer layers (epidermis, middle layer, and endothecium) and an inner cell layer (tapetum) surrounding the microsporocytes. Pollen production requires coordinated development of sporophytic and gametophytic tissues (Parish and Li, 2010; Chang et al., 2011; Hafidh et al., 2016; Fu et al., 2020). Haploid microspores are generated from pollen mother cells (PMC) through meiosis; then, two successive mitoses lead to the formation of binucleate and trinucleate mature pollen grains. During this process, the tapetum undergoes programmed cell death (PCD), providing the enzymes required for the release of the microspores from the tetrads, nutrients for pollen development and maturation and components for the pollen wall (Wilson and Zhang, 2009; Zhu et al., 2011; Zheng et al., 2020). Thus, the production of viable pollen grains requires a fine timing of tapetum degradation involving several key intracellular factors. Reactive oxygen species (ROS) have been shown to play an essential role in tapetum function and PCD in Arabidopsis and rice (Hu et al., 2011; Luo et al., 2013; Yi et al., 2016). For instance, high ROS levels in rice resulted in male sterility due to a delayed tapetum degradation (Hu et al., 2011; Luo et al., 2013), while low levels lead to early tapetum degradation (Xie et al., 2014). In both cases, the imbalance of ROS levels caused severe impairment of pollen development. Redox homeostasis is also critical for the regulation of different plant biological processes, including cell proliferation and differentiation (Zafra et al., 2010; Schippers et al., 2016; Huang et al., 2019; Lodde et al., 2021).

ROS are present in cells both in ionic and molecular states. Hydroxyl radical.OH and superoxide anion O_2_^⋅^^–^ represent the ionic state, while the molecular state consists mainly of singlet oxygen ^1^O_2_ and hydrogen peroxide H_2_O_2_. O_2_^⋅^^–^ is the precursor of different ROS and can be dismutated to H_2_O_2_ by the superoxide dismutase (SOD). Hydrogen peroxide is considered the most important ROS due to its high stability in the cell with a half-life of 10^–3^ s (Mittler, 2017; Mhamdi and van Breusegem, 2018). It can be transported through the cell membrane via aquaporins causing long-distance damage and participating in cell signaling (Miller et al., 2010; Lodde et al., 2021). ^⋅^OH is the most reactive ROS, and it can react with all biological molecules. However, it has a very short half-life, and thus, can only act locally at the cells where it is produced.

Cells have developed a diverse arsenal of mechanisms to deal with oxidative stress. ROS-scavenging mechanisms may be classified into two types: enzymatic and non-enzymatic. The enzymatic mechanisms rely on three main enzymes: superoxide dismutases (SOD), peroxidases and catalases (Arora et al., 2002; Huang et al., 2019; Lodde et al., 2021). The non-enzymatic mechanisms use low mass antioxidant molecules such as glutathione, ascorbic acid and flavonoids.

*Pisum sativum ENDOTHECIUM 1 (PsEND1*) is a pea (*Pisum sativum* L.) anther-specific gene displaying very early expression in the anther primordium and during anther development (Gómez et al., 2004). *PsEND1* promoter drives heterologous gene expression exclusively to the anthers, being equally active in dicots and monocots (Gómez et al., 2004; Roque et al., 2007, 2019; Briones et al., 2020). A −2.7 Kb region of *PsEND1* promoter has been used to drive the expression of the cytotoxic ribonuclease *barnase* gene (Hartley, 1988). *PsEND1* promoter was used to generate male-sterile plants by cell ablation of specific anther tissues, preventing the production of mature pollen grains in different ornamental (*Kalanchoe, Pelargonium*) and crops species (tomato, oilseed rape, tobacco, rice, wheat, poplar) (Roque et al., 2019; Briones et al., 2020). This ability makes *PsEND1* promoter a valuable tool for biotechnological applications, such as the production of hybrids, the elimination of allergenic pollen or gene containment.

*PsEND1* encodes a protein (25.7 KDa; Genbank accession number: AAM12036.1) containing four copies of a hemopexin-type conserved repeat (Beltrán et al., 2007). Hemopexins are heme-scavenging proteins preventing heme-associated oxidative stress in animals, fungi and plants. Heme is an electron transfer molecule of vital importance in several biological processes involving proteins associated with redox activity, such as peroxidases and hemopexins. However, free heme can be highly toxic for cells generating ROS (Vercellotti et al., 1994; Jeney et al., 2002; Kumar and Bandyopadhyay, 2005; Tolosano et al., 2010; Gáll et al., 2018). PsEND1 shows 72.17% sequence homology with the atypical pea storage protein PsPA2 (Higgins et al., 1987; Vigeoles et al., 2008; Robinson and Domoney, 2021) while it does not seem to have any homolog in *A. thaliana*. The role of hemopexins during anthers development has only been reported for the rice *OsHFP* gene (Chattopadhyay et al., 2015). It has been suggested that the heme-binding properties of OsHFP could regulate PCD in the anther tissues of rice through the regulation of ROS levels (Chattopadhyay et al., 2015).

In this work, we have functionally characterized the *PsEND1* gene of pea. Our findings suggest that *PsEND1* might be a target of MADS-domain transcription factors, playing an essential role during pollen development. The importance of this gene lies in its function as a ROS-scavenger during the differentiation of the anther tissues surrounding the microsporocytes.

## Materials and Methods

### Plant Material and Growth Conditions

*Arabidopsis thaliana* cv. Columbia plants were grown in the greenhouse under long-day conditions (16 h light/8 h dark) at 21°C. *Pisum sativum* cv. Bonneville, *Nicotiana benthamiana*, and *Nicotiana tabacum* cv. Xhanti plants were grown in the greenhouse under long-day conditions (16 h light/8 h dark) at 22°C (day) and 18°C (night).

### Promoter Sequence Analyses

*PsEND1* promoter region (Genbank accession number: AY324651) was analyzed in search for regulatory elements using the online software PLACE (Higo et al., 1999). We used the promoter region −986/−6, which is sufficient to direct the spatio-temporal expression of *PsEND1*.

### Promoter Cloning and Site-Directed Mutagenesis

The *PsEND1* promoter fragments were cloned in the plasmid pKGWFS7.0 and transcriptionally fused to the *uidA* reporter gene. Site-directed mutagenesis of the CArG-like motif was performed using the QuikChange II Site-directed mutagenesis kit (Agilent) according to the manufacturer instructions and using the primers END1mutF and END1mutR (Supplementary Table 1).

### GUS Staining

Floral tissues were infiltrated using three vacuum pulses of 5 min in GUS assay buffer [0.1 M NaH_2_PO_4_, 10 mM Na_2_EDTA.H_2_O, 0.5 M K_3_Fe(CN)_6_, 0.1% Triton X-100 and 0.3% 5-bromo-4-chloro-3-indolyl β-D-glucuronide (X-Gluc)] and incubated in this solution at 37°C for 16 h. Afterward, de-staining was carried out using successive washes with ethanol at 50, 70, and 90%. Subsequently, stained flowers were observed under a stereoscope (Leica MZ16F). GUS-positive zones were identified as those colored in blue.

### Subcellular Localization

The coding sequence of *PsEND1* was cloned in the plasmid pEarlyGate104 downstream the CaMV35S promoter and transcriptionally fused to the Yellow Fluorescent Protein (YFP). The construct was transformed into *Agrobacterium tumefaciens* strain C58C1. The transformed bacteria were used to transform leaves of *Nicotiana benthamiana* transiently. After 3 days, the leaves were infiltrated with an aniline blue solution (0.005% Aniline Blue in potassium phosphate buffer 70 mM, pH 9.0). Ten min later, fluorescence was detected in the leaves under a confocal microscope (AxioObserver 780, Zeiss).

### Prediction of PsEND1 3D Structure and Heme Binding Motifs

Prediction of PsEND1 3D structure was performed using the online tool swissmodel.expasy.org using the crystal structure of hemopexin fold protein CP4 from cowpea (SMTL ID: 3oyo.1; Gaur et al., 2011) as a template. The predicted 3D structure was visualized using the software Chimera (Pettersen et al., 2004) and the heme binding motifs using the online server HeMoQuest^1^ (George et al., 2020). This online interface detects transient heme binding nonapeptide motifs.

### Virus-Induced Gene Silencing

We used the plasmids pCAPE1 and pCAPE2 as vectors for gene silencing (Constantin et al., 2004). A fragment of the *PsEND1* coding sequence was amplified by PCR using the primers VIGSEND1F and VIGSEND1R (Supplementary Table 1) and cloned in the vector pCAPE2. The construct was transferred to the *Agrobacterium tumefaciens* strain C58C1. *Pisum sativum* cv. Bonneville 2-week-old plants were infiltrated as described by Constantin et al. (2004).

### Gene Expression Analyses by qRT-PCR

RNA was extracted using the E.Z.N.A.^®^ Plant RNA Kit (Omega Bio-tek) according to the manufacturer instructions. Two micrograms of total RNA were treated with DNase I (Thermo Scientific) following the manufacturer protocol. The first strand of cDNA was synthesized using 1 μg of treated RNA with the Primescript^TM^ RT-PCR kit (TAKARA, Tokyo, Japan). qRT-PCR was performed in a 7,500 Fast Real-time PCR System (Applied Biosystems, Foster City, CA, United States) using 20 ng of template cDNA mixed with EvaGreen^®^ Master Mix (Cultek, Madrid, Spain). For *PsEND1* expression analysis in the VIGS-*PsEND1* plants, we used the primers qEND1VIGSF and qEND1VIGSR (Supplementary Table 1). The constitutive gene *PsEF1* was used to normalize according to the 2^ΔΔCt^ method (Livak and Schmittgen, 2001).

### Pollen Viability Assay

Pollen was recovered from pre-dehiscent anthers under a stereomicroscope and incubated with Alexander stain at 50°C for 2 min (Alexander, 1969). The slides were later observed under an optical microscope (Leica DM5000).

### Overexpression of *PsEND1* in *Arabidopsis thaliana* and *Nicotiana tabacum*

The coding sequence of *PsEND1* was cloned downstream of the strong promoter CaMV35S in the plasmids pK2GW7. The construct was then transformed into the Agrobacterium strains C58C1 and LBA4404 to transform *A. thaliana* and *N. tabacum*, respectively. *A. thaliana* plants were transformed using the flower dip method (Zhang et al., 2006). Seeds of the transformed plants were recovered and germinated on a kanamycin selective medium. Tobacco plants were transformed as previously described (Hamza et al., 2018). Genomic DNA was extracted from the obtained plants using the E.Z.N.A.^®^ Plant DNA Kit (Omega Bio-tek). The integration of the transgenic DNA was checked by PCR using the primers PsEND1ATG and PsEND1STOP (Supplementary Table 1).

### Superoxide Anion Detection

In order to detect superoxide anion (O_2_^⋅^^–^) accumulation, Arabidopsis seedlings were immersed in a 0.2% w/v NBT solution in sodium phosphate buffer (pH 7.5). NBT reacts with superoxide anion forming a dark blue insoluble formazan compound. The seedlings were incubated overnight at room temperature, then the solution was discarded, and the seedlings were washed several times with 70% ethanol until complete removal of the chlorophyll.

### TdT-Mediated dUTP Nick-End Labeling Assay

TdT-mediated dUTP Nick-End Labeling (TUNEL) assay was performed with the DeadEND^TM^Fluoremetric TUNEL System kit (Promega) according to the manufacturer instructions. Samples were analyzed with a fluorescent microscope (Leica DM5000). Cells were stained with propidium iodide (1 μ/ml).

### Histological Sectioning

Flowers of *Pisum sativum* and *Arabidopsis thaliana* were fixed in formaldehyde/acetic acid/ethanol (10%:5%:50%). The flowers that were intended for the TUNEL assay were embedded in paraffin, while the rest of the samples were embedded in synthetic resin (Leica). The samples were later sectioned and stained either with 1% Toluidine Blue or with 1 μg/ml 2-(4-aminophenyl)-1H-indole-6-carboxamidine (DAPI). Toluidine blue stained sections were imaged by light microscopy, while DAPI stained slides were observed by fluorescence microscopy (Leica DM5000).

### Superoxide Dismutase and Peroxidase Activity

To measure SOD and PRX activity, pea flowers of each developmental stage were collected from different plants and mixed to form pools, as the level of gene silencing varies between plants. VIGS and control flowers were collected simultaneously. Frozen flowers of pea or leaves of *A. thaliana* and *N. tabacum* were ground in liquid nitrogen to a fine powder and homogenized in 500 μl of ice-cold extraction buffer (0.1 M Tris pH 7.0, 0.1% ascorbic acid, 0.1% L-cysteine, 0.5 M sucrose and 10 mg/ml PVP). The mixture was then centrifuged for 15 min at 4°C, and the supernatant was recovered. The total protein content of the crude extract was determined by the Bradford method (Bradford, 1976). The activity of SOD was determined using 5 μl of crude extract mixed with 200 μl of SOD buffer (PBS 50 mM pH 7.6, 0.1 mM EDTA, 50 mM sodium carbonate, 12 mM L-methionine, 10 μM riboflavin, 50 μM NBT in PBS 50 mM pH 7.6). The mixture was incubated for 10 min at room temperature under white light, and absorbance was measured at 560 nm. One unit of SOD activity is defined as the amount of enzyme required to inhibit 50% of the NBT photoreduction. PRX activity was measured adding 5 μl of crude extract to 200 μl PRX buffer (0.85 mM hydrogen peroxide in HEPES pH 7.0, 0.125 M 4-aminoantipyrene, 8.1 mg/ml phenol). The change in absorbance at 510 nm was measured for 2 min. Horseradish peroxidase at different known concentrations was used as a reference to generate a standard curve.

### Statistical Analyses

Statistical analyses were performed with the GraphPad Prism 9 software. ANOVA test was used to analyze the SOD and PRX activity assays in *A. thaliana* and *N. tabacum*, while *t*-test was used to compare enzymatic activity in pea flowers between the VIGS-*PsEND1* flowers and the control.

## Results

### Characterization and Functional Analysis of the *PsEND1* Promoter

**FIGURE 1:**
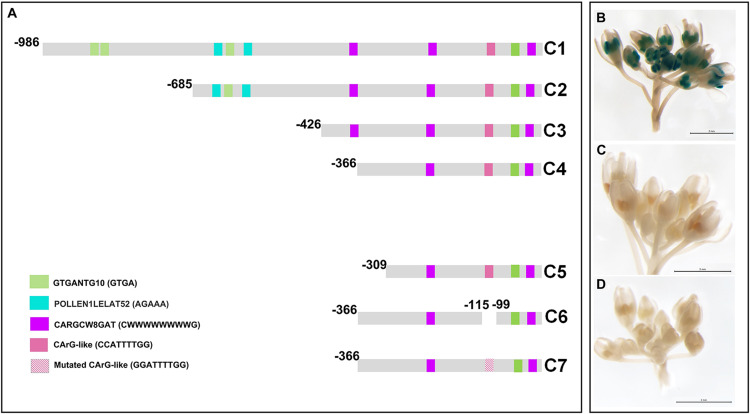
Deletion analysis of the *PsEND1* promoter. **(A)** Promoter regions used in the different constructs (C1–C7). The regulatory motifs related to the specific expression in anthers, GTGANTG10 and Pollen1lellat2, are highlighted in green and blue, respectively. CARG-like motifs are highlighted in pink and purple. **(B)** GUS staining of *Arabidopsis thaliana* flowers carrying the construct C4. **(C)** Gus staining of *A. thaliana* flowers carrying the construct C5. **(D)** Gus staining of *A. thaliana* flowers carrying the construct C7.

In a first approach, we have corroborated that the −986/−6 region of the *PsEND1* promoter is able to drive a strong anther-specific expression of the *uidA* (GUS) reporter gene in Arabidopsis. *In silico* analysis of this promoter region, using the online software PLACE, detected several putative regulatory motifs (Supplementary Table 2). Among these, we focused on the transcription factor binding DNA motifs associated to specific gene expression in anthers: GTGANTG10 (−799/−795; −794/−790; −693/−599; −597/−593; −81/−77) (Rogers et al., 2001) and Pollen1lellat2 (−607/−602; −551/−546) (Bate and Twell, 1998; Filichkin et al., 2004). In addition, we paid special interest to the DNA sequences recognized by transcription factors containing MADS-box domains, termed CArG motifs (Shore and Sharrocks, 1995; Riechmann and Meyerowitz, 1997; Folter and Angenent, 2006). We found three CARGCW8GAT motifs (Tang and Perry, 2003; CWWWWWWWWG at positions −375/−366; −247/−238; −57/−48 (Figure 1). We also included in our analysis a regulatory element previously described (Gómez et al., 2004) as a putative CArG-like motif (CCATTTTGG; −112/−104).

To identify the minimal promoter region sufficient to drive *PsEND1* anther-specific expression, we performed several promoter-reporter (*uidA*, GUS) constructs. The successive deletions contained the fragments −986/−6 (C1), −685/−6 (C2), −426/−6 (C3), −366/−6 (C4), and −309/−6 (C5) (Figure 1A). *Arabidopsis thaliana* plants were transformed with each of these constructs. The inflorescent apices from several primary (T0) transgenic plants were analyzed using flowers at different stages of development. The flowers of the Arabidopsis plants that contained the constructs C1 to C4 (Figure 1B) showed GUS staining in the anthers since the early stages of development. GUS staining was qualitatively high when all of these sequential deletions of the *PsEND1* promoter were used. However, no GUS signal was detected in the flowers of the transgenic plants transformed with the C5 construct (Figure 1C).

We tested whether the regulatory motif at position −112/−104 (CCATTTTGG), described by Gómez et al. (2004) as a putative CArG-like motif, might be critical for *PsEND1* gene expression. For this purpose, we generated a construct in which an internal fragment containing this motif was removed. The flowers of Arabidopsis plants harboring this construct did not show any GUS activity. This indicates that the CArG-like box motif (Gómez et al., 2004) present in the deleted fragment could be essential for *PsEND1* expression, at least within the context of the −366/−6 promoter. To confirm the importance of this regulatory element for the spatio-temporal expression pattern of the *PsEND1* gene, we performed a site-directed mutagenesis strategy. The CCATTTTGG sequence was converted into GGATTTTGG, thus lacking the conserved motif CArG. The Arabidopsis flowers harboring the mutated *PsEND1*promoter did not show any GUS staining in their anthers (Figure 1D). These findings confirm the importance of the putative CArG-like element for the regulation of *PsEND1*expression.

### Subcellular Localization of PsEND1

**FIGURE 2:**
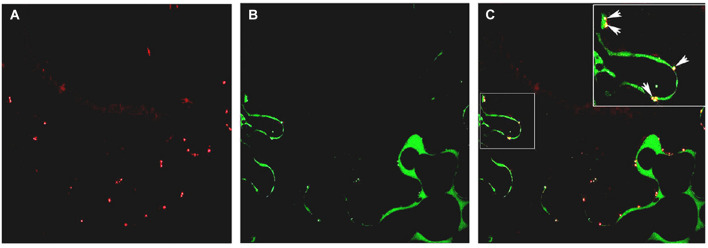
Subcellular localization of PsEND1 in *Nicotiana benthamiana* leaves as observed by confocal microscopy. **(A)** Plasmodesmata stained by aniline blue (red). **(B)** Localization of PsEND1 fused to YFP (green). **(C)** Co-localization of plasmodesmata and PsEND1. The yellow dots indicate the co-localization of both fluorescent signals.

In previous studies (Gómez et al., 2004), PsEND1 protein was immunolocalized in the anther tissues, but its subcellular localization was not determined. To investigate the subcellular localization of PsEND1protein, the *PsEND1* coding sequence was transcriptionally fused to the Yellow Fluorescent Protein (YFP). PsEND1 YFP-tagged protein was transiently expressed in *Nicotiana benthamiana* leaves. PsEND1 was detected by confocal microscopy in the cytoplasm and in plasmodesmata. Localization in plasmodesmata was confirmed by co-localization with aniline blue, a plasmodesmata marker (Figure 2).

### Prediction of PsEND1 3D Structure and Heme Binding Motifs

As previously described, PsEND1 presents four hemopexin domains (Supplementary Figure 2A; Beltrán et al., 2007). *In silico* 3D modeling based on the template of the cowpea, hemopexin fold protein showed that the hemopexin domains form a beta-propeller typical of hemopexins, surrounding a hollow shaft (Supplementary Figure 2B). This structure was described in different hemopexins (Paoli et al., 1999; Gaur et al., 2010; Chattopadhyay et al., 2012) and is thought to be responsible for binding heme groups (Paoli et al., 1999). Transient heme binding motifs were predicted using the online software HeMoQuest. Eight heme binding motifs were detected (Supplementary Figure 2C). These putative heme binding motifs were located on the PsEND1 3D model (Supplementary Figure 2D).

### Virus Induced Gene Silencing of *PsEND1* in Pea

**FIGURE 3:**
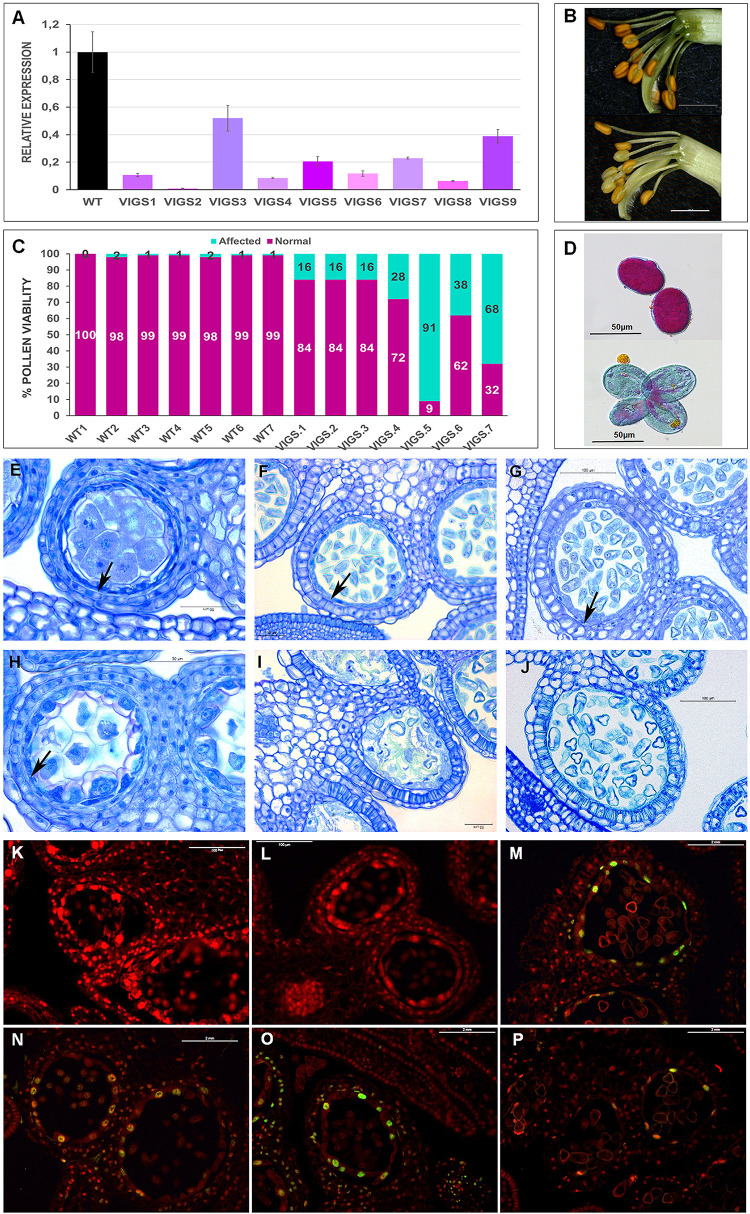
Phenotypic characterization of VIGS-*PsEND1* pea flowers. **(A)** Expression of *PsEND1* in flower buds of different VIGS plants. **(B)** Detail of the stamens of wild type (upper) and VIGS-*PsEND1* (bottom) plants. The anthers of the VIGS-*PsEND1* plants showed a white color and small size. **(C)** Percentage of viable pollen in the wild type and VIGS-*PsEND1* plants. **(D)** Alexander’s staining of pollen; wild type (upper) and VIGS plant (bottom). Viable pollen protoplasm is stained pink, while dead pollen is stained blue. Histological sections of wild type **(E–G)** and VIGS-*PsEND1* **(H–J)** pea flowers. **(E,H)** 3 mm flowers, **(F,I)** 5 mm flowers, **(G,J)** 7 mm flowers. Arrows in **(E–H)** indicate the presence of tapetal cells. Scale bars in **(E–J)** correspond to 100 μm. **(K–P)** Detection of DNA fragmentation by TUNEL assays of pea anthers of the wild type **(K–M)** and VIGS-*PsEND1* anthers **(N–P)**. Cell wall or membranes showed red fluorescence and the positive apoptotic nuclei stained with TUNEL were deep green.

To investigate the function of *PsEND1*, we generated pea plants with reduced levels of the gene, using Virus Induced Gene Silencing (VIGS) technology. As a negative control, we used the pCAPE1 vector. We analyzed nine independent VIGS-*PsEND1* lines and observed that the expression of *PsEND1* was highly reduced, ranging from 47 to 99% (Figure 3A). The majority of the flowers of the partially silenced plants presented a varying number of white anthers that appeared to contain few pollen grains (Figure 3B). Some anthers from silenced plants were also smaller in size. We recovered the pollen from control and VIGS-*PsEND1* plants and assessed its viability using Alexander’s stain (Alexander, 1969). Alexander’s stain colors aborted pollen grains blue-green and non-aborted pollen grains magenta-red. While the control plants showed over 98% viable pollen, the partially silenced plants contained between 16 and 91% aborted pollen (Figures 3C,D).

To further study the effect of *PsEND1*downregulation on pea anthers, we embedded flowers at different developmental stages and sizes in resin and performed histological cross-sections (Figures 3E–J). We observed that at the stage of 3 mm, the control anthers showed a thin layer of tapetal cells and round-shaped PMC where the cytoplasm is close to the membrane (Figure 3E). However, in the VIGS-*PsEND1* anthers, both the tapetal cells and PMC are swollen, and their cytoplasms shrunk (Figure 3H). At 5 mm stage, while in the control anthers, the pollen is normally formed and the tapetum is still present (Figure 3F), in the VIGS-*PsEND1* anthers, the tapetum has been degraded, and pollen grains seem to have collapsed and had been replaced by an amorphous mass (Figure 3I). Moreover, the endothecium has started to show secondary thickening and lignification, characteristics which were not observed in the control anthers. Later, at 7 mm size, the VIGS-*PsEND1* anthers presented no tapetal cells; the pollen was either collapsed or altered (Figure 3G), while the control still showing an intact tapetum and normal pollen cells (Figure 3J).

To analyze the effect of *PsEND1* gene silencing on tapetum development, we performed a TUNEL assay to visualize the cleavage of nuclear DNA (Figures 3K–P). In the VIGS-*PsEND1* anthers, we were able to detect chromatin fragmentation, the hallmark of PCD, in the tapetum of 5 mm flowers (Figure 3N). The fluorescent signal was maintained through stages of 6 mm (Figure 3O) and 7 mm (Figure 3P) in some locules. However, in the control anthers, no chromatin degradation could be detected at stages of 5 mm (Figure 3K) and 6 mm (Figure 3L). The first signal of PCD was detected in the 7 mm stage (Figure 3M). Therefore, while in the control anthers this process appears to be brief and initiated at late microspore state (7 mm flower), in the VIGS-*PsEND1* flowers, it seems to occur earlier and at a slow pace.

**FIGURE 4:**
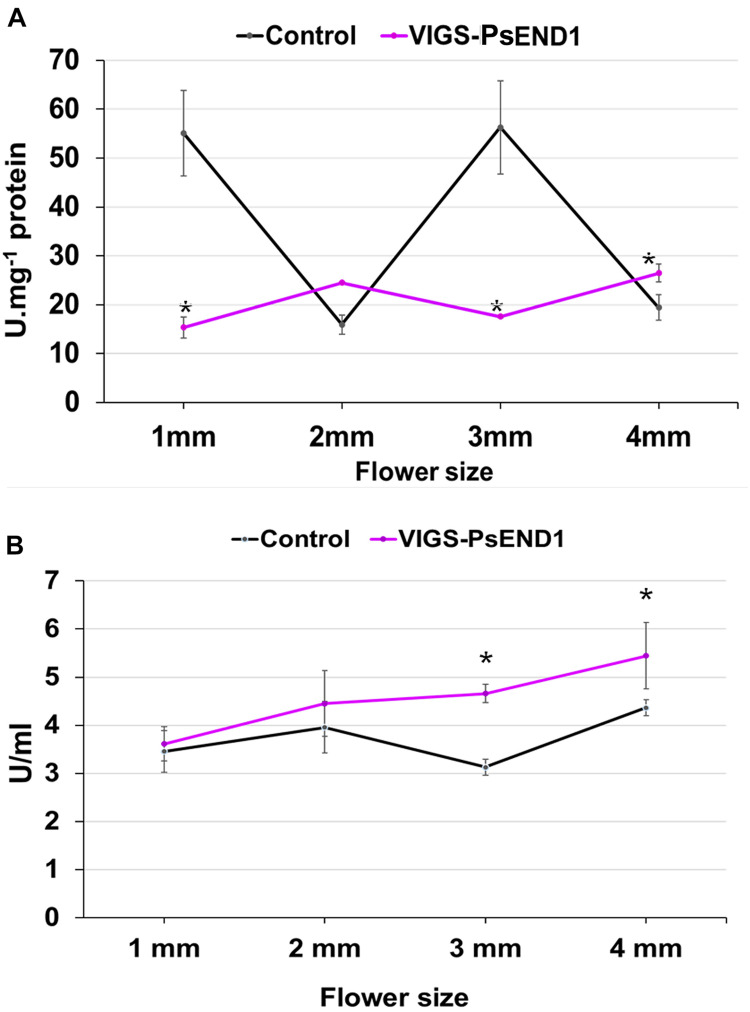
Superoxide dismutase and peroxidase activity in transgenic pea plants. **(A)** SOD activity in control and VIGS-*PsEND1* flowers at different developmental stages. SOD activity is significantly reduced in the VIGS-*PsEND1* flowers. **(B)** Peroxidase activity in control and VIGS-PsEND1 flowers at different developmental stages. At stages 3 and 4 mm, peroxidase activity increases in the VIGS-*PsEND1* plants compared to the control. *p ≤ 0.05, **p ≤ 0.01, ***p ≤ 0.001.

Different studies have shown that anther development and tapetum degeneration are highly sensitive to ROS balance (Hu et al., 2011; Xie et al., 2014; Yi et al., 2016; Jacobowitz et al., 2019; Zheng et al., 2019). ROS concentration in anther tissues is dynamic and finely regulated by several ROS-scavenging mechanisms in which participate enzymes such as peroxidases (PRX) and SOD. With the aim to investigate how *PsEND1* levels impact the enzymes involved in ROS regulation, we measured SOD and PRX activities in the VIGS-*PsEND1* pea flowers at four flower developmental stages (1–4 mm length flowers) (Figure 4). We found that in the control flowers, PRX and SOD activity follows antagonist patterns, varying during the development of the flower. When PRX activity increased (Figure 4B), SOD activity decreased (Figure 4A). In the VIGS-*PsEND1* flowers, at early stages, we observed a decreased SOD activity compared to the control, while PRX activity was increased. A dramatic reduction of SOD activity was observed at the flower sizes of 1 and 3 mm. While in the WT, SOD activity varied along developmental stages, in the VIGS-*PsEND1* flowers, the activity remains almost stable and low at the different flower developmental stages.

### Overexpression of *PsEND1* in *Arabidopsis thaliana* and *Nicotiana tabacum*

To further study *PsEND1* function, we expressed the gene under the control of the strong constitutive promoter CaMV35S in *Arabidopsis thaliana* and *Nicotiana tabacum*, two plant species containing no *PsEND1* homologs in their genomes.

**FIGURE 5:**
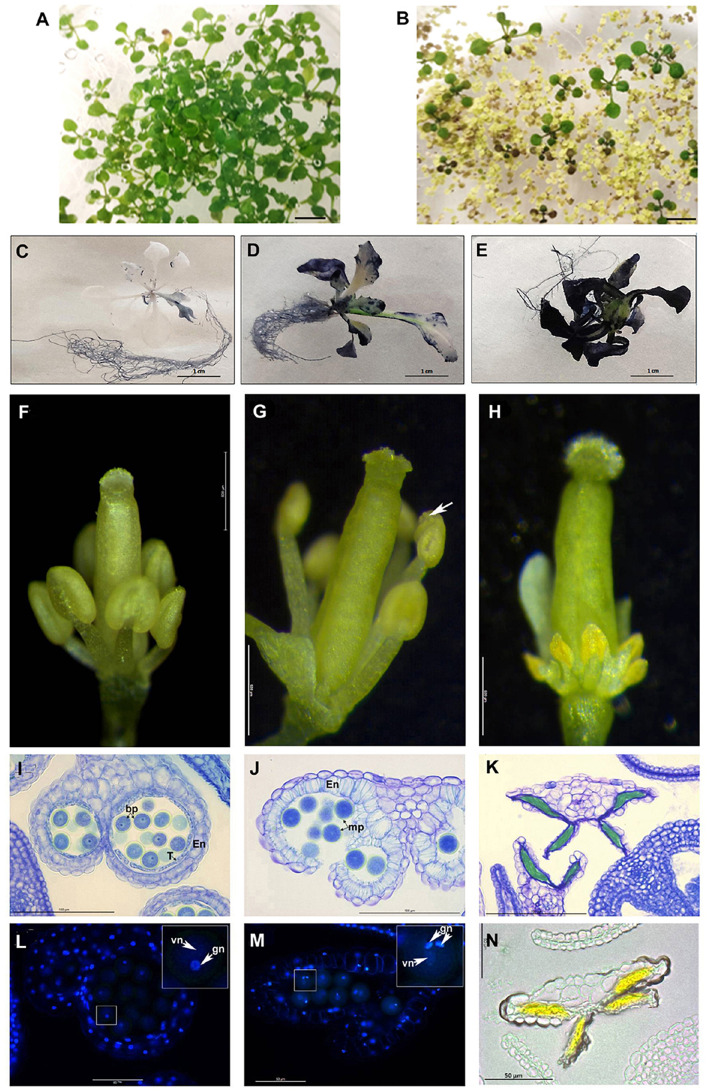
*PsEND1* ectopic expression in Arabidopsis plants. **(A–F)** Vegetative phenotype of 2-weeks-old Arabidopsis seedlings from the wild-type **(A)** and 35S:*PsEND1* plants **(B)**. **(C–E)** Detection of reactive oxygen species (ROS) production in *Arabidopsis thaliana* seedlings overexpressing *PsEN*D1 by Nitroblue tetrazolium (NBT) staining. **(F–H)** Floral phenotypes of *A. thaliana* plants overexpressing *PsEND1* (the petals and sepals have been removed). **(F)** Wild type flower. **(G)** 35S:*PsEND1* flower showing early dehiscence (white arrow). **(H)** 35S:*PsEND1* flower showing collapsed, arrow-shaped anthers**. (I–K)** Histological sections of anthers stained with toluidine blue. **(I)** Wild type flower anther showing a layer of intact tapetal cells and no endothecium secondary thickening. **(J)** Cross-section of a 35S:PsEND1 anther showing a disrupted septum and stomium secondary thickening of the endothecium and degenerated tapetum. **(K)** Cross-section of an arrow-shaped 35S:*PsEND1* anther where the pollen cells were replaced by an amorphous mass and the tapetal cell layer is absent. **(L–M)** Cross-section of wild type **(L)** and 35S:*PsEND1* anthers **(M)** of the same flower size, stained with DAPI and observed under a fluorescence microscope. **(L)** Cross-section of a wild type anther, presenting binucleate pollen (inset) and an intact tapetum. **(M)** Cross-section of a 35S:PsEND1 anther, with trinucleate pollen (inset) and no tapetal cell layer. **(N)** The collapsed locules of the 35S:PsEND1 anthers presenting an intense yellow colored amorphous mass. En, endotecium; T, tapetum; Bp, bicellular pollen; vn, vegetative nucleus; gn, generative nucleus. Bars in **(A,B)** represent 1 cm.

*A. thaliana* transformed seeds were grown on selective media. After germination, some seedlings developed to a similar size to the control ones, while others remained small and presented a dark color (Figures 5A,B). To investigate if these changes might be due to ROS accumulation, we stained the seedlings with NBT. The overexpressing plants showed an accumulation of superoxide radical O_2_^⋅^^–^ as shown by the intense staining of the rosette leaves (Figures 5C–E). It is noteworthy to mention that the most affected plants in size and development presented the highest O_2_^⋅^^–^ levels (Figures 5D,E). Several transgenic plants were later acclimatized in the greenhouse, although the smaller plants did not grow and died. The rest of the plants managed to grow but were smaller in size than the control ones.

The flowers of the 35S:*PsEND1* plants showed two types of developmental anther defects that coexist in the same plants: early anther dehiscence or complete anther collapse. In pre-anthesis flowers, at an early stage where the stamens were still shorter than the style, some anthers were already dehiscent (Figure 5G). The second type corresponds to flowers that showed collapsed stamens, with short filaments and arrow-shaped anthers with an intense yellow color (Figure 5H). These flowers were embedded in resin and stained with toluidine blue to visualize the effect on the internal structure of the anthers (Figures 5I–K). The histological analysis confirmed the macroscopic observations. In the anthers that seemed to have precocious dehiscence, the septum and the stomium have already been disrupted, thus liberating the pollen (Figure 5J). Moreover, the endothecium showed secondary thickening, and the tapetum was absent. Mutant flowers were compared to the wild type ones (Figure 5I) at the same stage. The developmental stage of the flowers was determined by the development of the carpel. The floral sections were subsequently stained with DAPI. We observed that the pollen grains of the dehiscent transformed anthers were mature and trinucleate (Figure 5M), while the pollen grains of the control flowers were still binucleate (Figure 5N). The anthers of transgenic flowers had an accelerated development leading to early dehiscence and pollen maturation. Arrow-shaped anthers of transgenic flowers were also sectioned and observed. Their locules were surrounded by a single cell layer corresponding to the epidermis, while the endothecium and the tapetum were absent. Inside the locules, the anthers presented an intense yellow colored amorphous mass instead of the pollen cells (Figures 5K,N), probably due to an accumulation of flavonoids. At early stages, the anthers were similar to the wild type, being the first alterations observed at the stage of meiocytes, previous to the formation of tetrads. Indeed, tetrads could not be observed in the more affected anthers; the last stage observed being the meiocyte one. After this stage, the PMC and the tapetum degenerated (Supplementary Figure 1).

**FIGURE 6:**
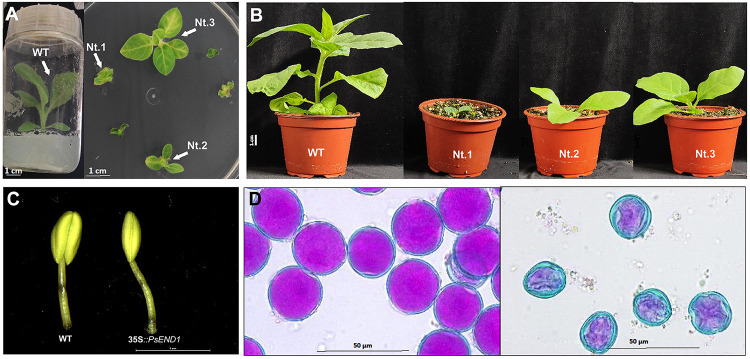
Phenotypes of transgenic tobacco plants overexpressing *PsEND1.* **(A)** Tobacco plants 8 weeks after transformation. The control plant (left) has elongated and rooted and are ready to be acclimatized. The transgenic plants (right) are still much smaller and have not elongated nor rooted, yet. **(B)** Wild type (WT) and transgenic (Nt1, Nt2, and Nt3) tobacco plants 1 week after acclimatization in the greenhouse. The transgenic plants are smaller than the control. **(C)** Anthers of the transgenic plants (right) are smaller and lack two locules. **(D)** Alexander’s staining of pollen grains from the control (left) and transgenic tobacco plants (right). Viable pollen is round-shaped and stained in pink.

We also overexpressed *PsEND1* in *Nicotiana tabacum*. Only a few plantlets were regenerated because most of the calli failed to differentiate. Most of them remained small and did not succeed to root (Figure 6A). Three plants were acclimatized in the greenhouse. Similarly to what was observed with *A. thaliana* plants, these transgenic tobacco plants were smaller than the control ones (Figure 6B), and some of the transgenic flowers presented smaller anthers or anthers formed by only two locules (Figure 6C). We recovered the pollen of these anthers and stained it with Alexander’s stain. We found that this pollen was non-viable (Figure 6D).

**FIGURE 7:**
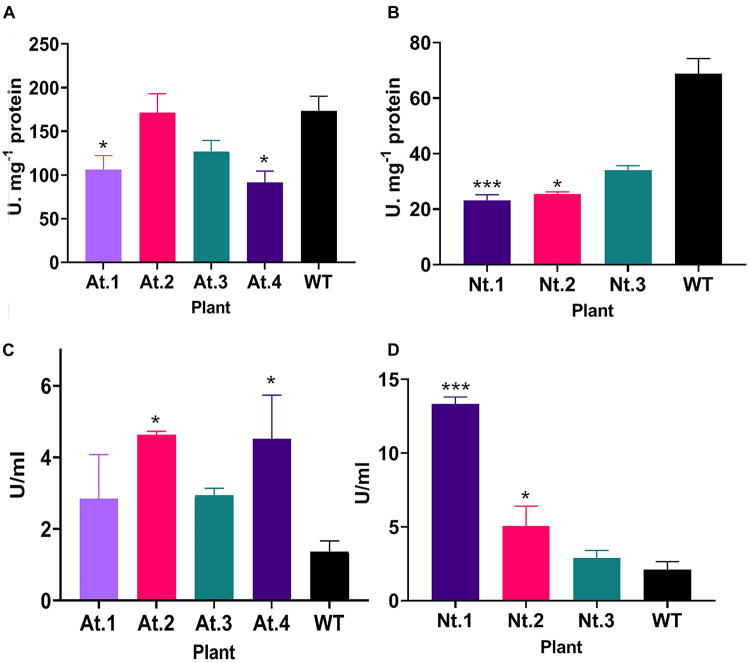
Superoxide dismutase and peroxidase activity in transgenic *Arabidopsis thaliana* and *Nicotiana tabacum* plants. **(A)** SOD activity in transgenic *A. thaliana* plants. Plants At.1 and At.4 show a significant decrease in SOD activity compared to the wild type. **(B)** SOD activity in transgenic *N. tabacum* plants. The transgenic plants Nt.1 and Nt.2 show a significant decrease in SOD activity compared to the wild type. **(C)** Peroxidase activity in transgenic *A. thaliana* plants. Plants At.2 and At.4 show a significant increase in peroxidase activity compared to the wild type. **(D)** Peroxidase activity in transgenic *N. tabacum* plants. The transgenic plants Nt.1 and Nt.2 show a significant increase in peroxidase activity compared to the wild type. *p ≤ 0.05, **p ≤ 0.01, ***p ≤ 0.001.

We then analyzed SOD and peroxidase activity in the rosette of *A. thaliana* and the leaves of N. *tabacum* overexpressing *PsEND1*. Similarly, in the overexpressing transgenic Arabidopsis and tobacco plants, SOD activity (Figures 7A,B) decreased, and PRX activity (Figures 7C,D) increased. This effect was stronger in the tobacco plant Nt.1, which was the most altered in size.

## Discussion

### The Hemopexin-Like Protein PsEND1 Is a Putative Target of MADS-Box Transcription Factors

The *PsEND1* promoter deletion analysis showed in this work indicates that a region of approximately 0.3 kb (−366 to −6) upstream of the transcriptional start site contains the PsEND1 minimal promoter. Deleted sequences between C4 and C5 constructs (from −336 to −309) comprises different TF-binding sites as well as different common regulatory elements to eukaryotes and plants, such as the DOF (DOFCOREZM) (Yanagisawa and Schmidt, 1999; Yanagisawa, 2000) and MYC (MYCCONSENSUSAT) (Chinnusamy et al., 2003, 2004; Oh et al., 2005). These TFs binding elements seem to be essential for *PsEND1* regulation in the context of the −366 to −6 domain. This defined minimal promoter region (−366 to −6) contains a transcription binding DNA motif associated with the specific gene expression in anthers (GTGANTG10) (Rogers et al., 2001), two CARGCW8GAT motifs (Tang and Perry, 2003; Folter and Angenent, 2006) and a putative CArG-like motif (Gómez et al., 2004; Figure 1A). The absence of GUS staining in the anthers of the Arabidopsis plants containing the construct C5 (−309 to −6) may be explained by a possible cooperative protein-protein interaction between MADS-domain proteins with other transcription factors. One of the CARGCW8GAT (−247 to −238) motifs falls in the vicinity of the removed TF binding sites from −366 to −309. It has been suggested that the presence of other transcription factor binding sites in the proximity of CArG-box motifs could be essential for MADS-domain proteins interactions and, consequently, target gene regulation (Aerts et al., 2018).

We demonstrated that the CArG-like regulatory motif present at the position −112 to −104 (CCATTTTGG; Gómez et al., 2004) is essential to confer promoter activity in developing anthers. MADS-box proteins possess similar DNA-binding specificities, although some differences between each protein exist in this regard (Folter and Angenent, 2006). This DNA-binding site is termed CArG motif with the overall consensus CC(A/T)_6_GG (Shore and Sharrocks, 1995; Riechmann and Meyerowitz, 1997; Folter and Angenent, 2006), recently renamed perfect CArG-box (Aerts et al., 2018). Several variants of this sequence have been recognized as regulatory motifs for binding to some MADS-domain proteins (Folter and Angenent, 2006; Aerts et al., 2018). These motifs still conserve the DNA-binding properties of the MADS domain proteins as long as the mismatches do not eliminate either the C or G sequences flanking the A/T core. The A/T core can include C or G and may be formed by six to eight nucleotides (Gómez-Mena et al., 2005; Folter and Angenent, 2006). Besides, it has been reported that the most chosen DNA binding motif of MADS-box proteins was a CArG-box with an NAA extension comparing with a perfect CArG-box (Folter and Angenent, 2006; Aerts et al., 2018). The sequences CCATTTTGG found in the 5’ *PsEND1* sequence is an almost perfect CArG motif. It features the typical dyad symmetry ending as CC and GG but differs from the rest since it has five nucleotides in the A/T core instead of six. However, this CArG-like regulatory motif (Gómez et al., 2004) contains an extension NAA: CC(CA/T)_5_GGGAA at position 13. CArG-box has 10 positions, being the first C, the position 1 and the last G, the defined position 10 (Aerts et al., 2018). It has been demonstrated that there is a preference for adenines at positions 12 and 13, thus extending the consensus motif of a perfect CArG-box to CC(A/T)_6_GGNAA (Aerts et al., 2018). Therefore, the sequence CC(CA/T)_5_GGGAA found in the 5′ region of the pea *PsEND1* gene might be a new CArG-box regulatory motif variant.

In summary, the flexibility of the CArG-box sequences recognized by MADS target genes, the early specific expression of *PsEND1* in the anther primordia and the results showed in this work suggest that *PsEND1* could be a target of the MADS-domain proteins that specify the stamen identity in *Pisum sativum*. Further experiments will be needed to confirm that the Pisum MADS-box proteins that confer stamen identity can interact with this putative CArG-box regulatory motif. Interestingly, the rice hemopexin fold protein gene (*OsHFP*) promoter also presents a CArG-like regulatory element essential for its specific expression in anthers (Chattopadhyay et al., 2012). In line with this, the C-function MADS-box gene *MADS3* has been related to the promoter of a ROS-scavenging gene in rice (Hu et al., 2011). Accordingly, our data suggest that *PsEND1* could be a hemopexin regulated by a MADS-box protein, modulating redox homeostasis during early anther development in pea.

### *PsEND1* Is Essential for Proper Pollen Development in *Pisum sativum*

In this work, we have shown that *PsEND1* is of major importance for the development of pollen and the surrounding tissues. *PsEND1* expression begins at an early stage of anther development, in the stamen primordium. Later, it is expressed in the primary parietal cells, precursor cells of the endothecium, middle layer, and tapetum tissues (Gómez et al., 2004). At this stage, these cells are contiguous with the primary sporogenous cell lineage, and *PsEND1* is participating in the modulation of ROS in the cells that will differentiate into different sporophytic tissues, including the tapetum. Therefore, its downregulation affects the development and degeneration of the tapetal cells and causes the abortion of pollen. The tapetum is a transitory apoptotic tissue that provides nutrients to microspores. Hence, a functional tapetum is essential for the proper development of microspores. In fact, different male-sterile mutants present tapetum ablation or affected tapetum (Mariani et al., 1990; Goldberg et al., 1993; Sanders et al., 1999; Kawanabe et al., 2006; Parish and Li, 2010; García et al., 2017). Tapetum PCD occurs at late microsporogenesis stages. The precise timing of this process is of vital importance for pollen development (Ko et al., 2017; Sun et al., 2018; Bai et al., 2019; Gao et al., 2019; Shukla et al., 2019; Xu et al., 2019; Zheng et al., 2019; Lei and Liu, 2020; Mondol et al., 2020). In this context, different studies have demonstrated that the arrest of tapetum development at early stages results in meiotic cell cycle arrest and meiocytes maturation failure (Murmu et al., 2010; Cui et al., 2018). Accordingly, our findings show that *PsEND1* plays an essential role in the synchronization of tapetum degeneration and, thus, in the development of viable pollen grains in *Pisum sativum*.

### *PsEND1* Participates in Pollen Development Through the Modulation of Redox Homeostasis

Through *in silico* 3D modeling, we have shown that PsEND1 presents the typical beta-propeller structure responsible for heme binding in the hemopexins (Paoli et al., 1999). The presence of eight possible heme binding motifs in the PsEND1 protein has been predicted using the online HeMoQuest software, which shows a high level of accuracy (92%) in identifying heme binding motifs (George et al., 2020). Taken together, these results suggest that PsEND1 possesses the biochemical capacity of binding heme and, consequently, could participate in redox modulation.

The overexpression of *PsEND1* in Arabidopsis seedlings produces an accumulation of superoxide anion O_2_^⋅^^–^ supporting its implication in ROS modulation. Previous studies have linked heme with ROS scavenging enzymes. For instance, in *Saccahromyces cerevisiae* it had been shown that heme regulates the expression of SOD (Pinkham et al., 1997). Moreover, it has been demonstrated that the expression of the rice Hemopexin *OsHFP* in *E. coli* affects the expression of a SOD isozyme (Chattopadhyay et al., 2015). Accordingly, the downregulation of *PsEND1* in pea and its ectopic overexpression in *A. thaliana* and tobacco affected the enzymatic activities of SOD and peroxidase. Additionally, Arabidopsis plants overexpressing *PsEND1* accumulated flavonoids in their anthers, which are also ROS-scavenging molecules. This confirms that *PsEND1* interferes with different ROS-scavenging mechanisms involved in the maintenance of balanced redox levels. ROS have been shown to play an essential role in tapetum development and PCD in model dicots and rice (Hu et al., 2011; Xie et al., 2014; Yi et al., 2016; Jacobowitz et al., 2019; Zheng et al., 2019). Several studies have shown that the loss of redox balance, either by an increase or a decrease in ROS, impairs tapetum degradation and pollen development (Hu et al., 2011; Luo et al., 2013; Xie et al., 2014). For instance, in tomato (Yan et al., 2020) and rice (Yi et al., 2016) it has been found that a decrease in ROS levels caused a delay in tapetum degradation. Nonetheless, in wheat, an excess of ROS levels has been associated with delayed tapetum degeneration (Liu et al., 2018). These studies are in agreement with the anther defects observed here by downregulation or overexpression of *PsEND1* in different species.

In Arabidopsis, it has been shown that the apoplastic class III peroxidases (PRX9 and PRX40) are required for the correct development of the anthers. *prx9-1* and *prx40-2* mutants showed defects similar to those observed in the VIGS-*PsEND1* anthers, with swollen tapetal cells at early meiosis stages and aborted pollen (Jacobowitz et al., 2019). It is also noteworthy that this class of peroxidases have also been found in pollen cells and, therefore, could play an essential role in their development (Sankaranarayanan et al., 2020). In tobacco and tomato plants, the manipulation of the expression of Rbohs proteins, which are involved in ROS regulation, resulted in the impairment of tapetal degradation and pollen development. Similarly over-accumulation of ROS in rice anthers generated premature initiation of tapetum PCD and pollen abortion (Zheng et al., 2019). These results are in accordance with the phenotype observed in the VIGS-*PsEND1* anthers where we detected by TUNEL assay the early degradation of the tapetum cells, leading to pollen abortion.

It is noteworthy that in the pea VIGS-*PsEND1* and *A. thaliana* 35S:*PsEND1* flowers, the first defects in pollen are observed approximately at the same stage: when the PMC are undergoing the first meiosis. Indeed, ROS have been suggested, for a long time, to play a role in cell cycle progression as an intrinsic signal (Ho and Dowdy, 2002; Jorgensen and Tyers, 2004). For instance, Reichheld et al. (1999) have shown that oxidative stress affect cell divisions and SOD expression in *Nicotiana tabacum* both in plant cell culture and plants. Moreover, it has been shown that in Arabidopsis embryogenic roots, the G1 phase is accelerated in the oxidized state and slowed in the reduced state (de Simone et al., 2017). Our findings are in accordance with these results. Under the oxidative stress caused by the overexpression of *PsEND1* in Arabidopsis and after the early meiosis stage, the PMC and surrounding tissues (tapetum and endothecium) either degenerated and collapsed or presented an accelerated development leading to early pollen maturation, tapetum degradation and endothecium lignification. In plants, studies have shown that there is a tightly regulated gradient of ROS during the development of the pollen and throughout the meiosis (Zafra et al., 2010; Xu et al., 2019). In pea, the regulation of ROS and the activity of antioxidant enzymes in flowers have not been described previously. In our work, we determined the activity of two of the major ROS detoxifying enzymes: SOD and PRX, during the early anther developmental stages. Interestingly, the activity of both enzymes is altered at the same stage in the VIGS-*PsEND1* flowers, where the depletory effects on tapetum and PMC are observed (stage 3 mm). These findings confirm that the fine quantitative and spatio-temporal regulation of redox homeostasis at early anther developmental stages and, especially at the start of meiosis, is critical for the correct development of anther tissues and proper pollen development.

Subcellular localization of hemopexins has not been described in plants before. Our work is the first to report the localization of a plant hemopexin in plasmodesmata. These channels allow the communication between neighboring cells as well as distantly located cells through the symplastic pathway (Ding et al., 2003; Cilia and Jackson, 2004; Heinlein and Epel, 2004). They are responsible for transporting non-cell-autonomous signaling molecules such as transcription factors, small RNAs or ROS (Haywood et al., 2002; Heinlein, 2002; Wu et al., 2002; Sager and Lee, 2018). Also, due to their proximity to the cell wall, it has been suggested by previous studies that plasmodesmata act as a bridge between the symplastic and apoplastic compartments (Stahl and Simon, 2013). In plants, peroxidases are heme-containing enzymes that have been found in the apoplast, where they participate in redox homeostasis. PsEND1 localization suggests that this protein might interact directly or indirectly with peroxidases to modulate ROS levels and transport through plasmodesmata.

During early pollen development stages, plasmodesmata exist at the junction of different tissues: middle layer-tapetum, between tapetum cells, tapetum- PMC and between pre-meiotic PMC (Steer, 1977; Radice et al., 2008; Sager and Lee, 2014). This connection permits the feeding of the developing PMC by the tapetum. This would explain the effect of *PsEND1* expression alteration on tapetum and pollen cells even though the gene is not expressed in these cells. H_2_O_2_, which is produced in the apoplast by dismutation of O_2_^⋅^^–^, is the most stable ROS, with a half-life of 10^–3^ s (Mittler, 2017; Mhamdi and van Breusegem, 2018). Then, it can be transported through the plasma membrane and participate in cell signaling or cause long-distance damage (Bienert et al., 2006; Lodde et al., 2021). *PsEND1* is expressed since very early developmental stages of the anthers in the cell layers neighboring those that will produce the tapetum and pollen. Thus, *PsEND1* might be participating in the communication among cell layers modulating ROS accumulation during the early stages of anther development.

Taken together, our results indicate that *PsEND1* is a central player in the maintenance of balanced redox levels during anther development. Further studies with fine monitoring of heme levels, ROS and oxidants production would provide further insight into the biochemical mechanisms by which *PsEND1* modulates redox homeostasis in the anthers.

## Data Availability Statement

The original contributions presented in the study are included in the article/Supplementary Material, further inquiries can be directed to the corresponding author/s.

## Author Contributions

RH, ER, and CG-M performed the experiments. RH, ER, CG-M, FM, JB, and LC conceived the experiments and analyzed the data. LC and CG-M wrote the grants that funded this work. RH, ER, and LC wrote the manuscript. All authors read and approved the final version of this manuscript.

## Conflict of Interest

The authors declare that the research was conducted in the absence of any commercial or financial relationships that could be construed as a potential conflict of interest.

## Publisher’s Note

All claims expressed in this article are solely those of the authors and do not necessarily represent those of their affiliated organizations, or those of the publisher, the editors and the reviewers. Any product that may be evaluated in this article, or claim that may be made by its manufacturer, is not guaranteed or endorsed by the publisher.

## Footnotes

1
http://131.220.139.55/SeqDHBM/

## Supplementary Material

Supplementary Figure 1 Comparative cross-sections of WT and 35S:*PsEND1 Arabidopsis thaliana* flowers stained with toluidine blue. **(A)** Wild type; **(B,C)** 35S:*PsEND1.* At the first stages of development, we do not observe differences between anthers in **(A,B)**, while at the stage of meiocytes **(C)** most of the anthers have collapsed. After this stage, the pollen mother cells and the tapetum degenerate.

Supplementary Figure 2 PsEND1 sequence and predicted 3D structure showing the heme binding sites. **(A)** PsEND1 sequence, the hemopexin motifs are highlighted in blue boxes. **(B)** Predicted PsEND1 3D structure. Cyan: Hemopexin domain at position 4–55; Pink: Hemopexin domain at position 62–112; Orange: Hemopexin domain at position 118–165; Green: Hemopexin domain at position 171–222. **(C)** Heme binding motifs predicted by the software HeMoQuest. **(D)** PsEND1 3D model showing the localization of eight heme binding motifs predicted by the software HeMoQuest. The predicted heme binding sites are represented by spheres of different colors.

## References

[B1] Aerts N. de Bruijn S. van Mourik H. Angenent G. C. van Dijk A. D. (2018). Comparative analysis of binding patterns of MADS-domain proteins in *Arabidopsis thaliana*. *BMC Plant Biol.* 18:131. 10.1186/s12870-018-1348-8 29940855 PMC6019531

[B2] Alexander M. P. (1969). Differential staining of aborted and non aborted pollen. *Stain Technol.* 44 117–122. 10.3109/10520296909063335 4181665

[B3] Arora A. Sairam R. K. Srivastava G. C. (2002). Oxidative stress and antioxidative system in plants. *Curr. Sci.* 82 1227–1238.

[B4] Bai W. Wang P. Hong J. Kong W. Xiao Y. Yu X. (2019). *Earlier Degraded Tapetum1* (*EDT1*) encodes an ATP-citrate lyase required for tapetum programmed cell death. *Plant Physiol.* 181 1223–1238. 10.1104/pp.19.00202 31515447 PMC6836821

[B5] Bate N. Twell D. (1998). Functional architecture of a late pollen promoter: pollen-specific transcription is developmentally regulated by multiple stage-specific and co-dependent activator elements. *Plant Mol. Biol.* 37 859–869. 9678581 10.1023/a:1006095023050

[B6] Beltrán J. P. Roque E. Medina M. Madueño F. Gómez M. D. Cañas L. A. (2007). Androesterilidad inducida mediante ingeniería genética en plantas: fundamentos y aplicaciones biotecnológicas. *Anal. Real Acad. Nacional Farmacia* 73 1237–1264.

[B7] Bienert G. P. Schjoerring J. K. Jahn T. P. (2006). Membrane transport of hydrogen peroxide. *Biochem. Biophys. Acta* 1758 994–1003. 16566894 10.1016/j.bbamem.2006.02.015

[B8] Bradford M. M. (1976). A rapid and sensitive method for the quantitation of microgram quantities of protein utilizing the principle of protein-dye binding. *Analyt. Biochem.* 72 248–254. 942051 10.1016/0003-2697(76)90527-3

[B9] Briones M. V. Hoenicka H. Cañas L. A. Beltrán J. P. Hanelt D. Sharry S. (2020). Efficient evaluation of a gene containment system for poplar through early flowering induction. *Plant Cell Rep.* 39 577–587. 32052127 10.1007/s00299-020-02515-1 PMC7165154

[B10] Chang F. Wang Y. Wang S. Ma H. (2011). Molecular control of microsporogenesis in Arabidopsis. *Curr. Opin. Plant Biol.* 14 66–73. 10.1016/j.pbi.2010.11.001 21145279

[B11] Chattopadhyay T. Bhattacharyya S. Das A. K. Maiti M. K. (2012). A structurally novel hemopexin fold protein of rice plays role in chlorophyll degradation. *Biochem. Biophys. Res. Commun.* 420 862–868. 10.1016/j.bbrc.2012.03.089 22465006

[B12] Chattopadhyay T. Das P. K. Roy S. Maiti M. K. (2015). Proposed physiological mode of action of rice hemopexin fold protein OsHFP: linking heme-binding with plant cell death. *Acta Physiol. Plantarum* 37:95.

[B13] Chinnusamy V. Ohta M. Kanrar S. Lee B. H. Hong X. Agarwal M. (2003). ICE1: a regulator of cold-induced transcriptome and freezing tolerance in *Arabidopsis*. *Genes Dev.* 17 1043–1054. 10.1101/gad.1077503 12672693 PMC196034

[B14] Chinnusamy V. Schumaker K. Zhu J. K. (2004). Molecular genetic perspectives on cross-talk and specificity in abiotic stress signalling in plants. *J. Exper. Bot.* 55 225–236. 10.1093/jxb/erh005 14673035

[B15] Cilia M. L. Jackson D. (2004). Plasmodesmata form and function. *Curr. Opin. Cell Biol.* 16 500–506. 10.1016/j.ceb.2004.08.002 15363799

[B16] Constantin G. D. Krath B. N. MacFarlane S. A. Nicolaisen M. Johansen I. E. Lund O. S. (2004). Virus-induced gene silencing as a tool for functional genomics in a legume species. *Plant J.* 40 622–631. 10.1111/j.1365-313x.2004.02233.x 15500476

[B17] Cui Y. Hu C. Zhu Y. Cheng K. Li X. Wei Z. (2018). CIK receptor kinases determine cell fate specification during early anther development in *Arabidopsis*. *Plant Cell* 30 2383–2401. 10.1105/tpc.17.00586 30201822 PMC6241272

[B18] Folter S. Angenent G. C. (2006). Trans meets cis in MADS science. *Trends Plant Sci.* 11 224–231. 10.1016/j.tplants.2006.03.008 16616581

[B19] de Simone A. Hubbard R. De La Torre N. V. Velappan Y. Wilson M. Considine M. J. (2017). Redox changes during the cell cycle in the embryonic root meristem of Arabidopsis thaliana. *Antioxid. Redox Signal.* 27 1505–1519. 10.1089/ars.2016.6959 28457165 PMC5678362

[B20] Ding B. Itaya A. Qi Y. (2003). Symplasmic protein and RNA traffic: regulatory points and regulatory factors. *Curr. Opin. Plant Biol.* 6 596–602. 14611959 10.1016/j.pbi.2003.09.010

[B21] Filichkin S. A. Leonard J. M. Monteros A. Liu P. P. Nonogaki H. (2004). A novel endo-β-mannanase gene in tomato LeMAN5 is associated with anther and pollen development. *Plant Physiol.* 134 1080–1087. 14976239 10.1104/pp.103.035998 PMC389932

[B22] Fu Y. Li M. Zhang S. Yang Q. Zhu E. You C. (2020). Analyses of functional conservation and divergence reveal requirement of bHLH010/089/091 for pollen development at elevated temperature in *Arabidopsis*. *J. Genet. Genom.* 47 477–492. 10.1016/j.jgg.2020.09.001 33393464

[B23] Gáll T. Pethő D. Nagy A. Hendrik Z. Méhes G. Potor L. (2018). Heme induces endoplasmic reticulum stress (HIER stress) in human aortic smooth muscle cells. *Front. Plant Sci.* 9:1595. 10.3389/fphys.2018.01595 30515102 PMC6255930

[B24] Gao J. Li Q. Wang N. Tao B. Wen J. Yi B. (2019). Tapetal expression of BnaC. MAGL8. a causes male sterility in Arabidopsis. *Front. Plant Sci.* 10:763. 10.3389/fpls.2019.00763 31249581 PMC6582705

[B25] García C. C. Nepi M. Pacini E. (2017). It is a matter of timing: asynchrony during pollen development and its consequences on pollen performance in angiosperms-a review. *Protoplasma* 254 57–73. 10.1007/s00709-016-0950-6 26872476

[B26] Gaur V. Chanana V. Jain A. Salunke D. M. (2011). The structure of a haemopexin-fold protein from cow pea (*Vigna unguiculata*) suggests functional diversity of haemopexins in plants. *Acta Crystallogr. Sect. F Struct. Biol. Cryst. Commun.* 67 193–200. 10.1107/s1744309110051250 21301085 PMC3034607

[B27] Gaur V. Qureshi I. A. Singh A. Chanana V. Salunke D. M. (2010). Crystal structure and functional insights of hemopexin fold protein from grass pea. *Plant Physiol.* 152 1842–1850. 10.1104/pp.109.150680 20147493 PMC2850029

[B28] George A. A. P. Lacerda M. Syllwasschy B. F. Hopp M.-T. Wißbrock A. Imhof D. (2020). HeMoQuest: a webserver for qualitative prediction of transient heme binding to protein motifs. *BMC Bioinform.* 21:124. 10.1186/s12859-020-3420-2 32216745 PMC7099796

[B29] Goldberg R. B. Beals T. P. Sanders P. M. (1993). Anther development: basic principles and practical applications. *Plant Cell* 5:1217. 10.2307/3869775 PMC160355 8281038

[B30] Gómez M. D. Beltrán J. P. Cañas L. A. (2004). The pea END1 promoter drives anther-specific gene expression in different plant species. *Planta* 219 967–981. 10.1007/s00425-004-1300-z 15221384

[B31] Gómez-Mena C. de Folter S. Costa M. M. R. Angenent G. C. Sablowski R. (2005). Transcriptional program controlled by the floral homeotic gene *AGAMOUS* during early organogenesis. *Development* 132 429–438. 10.1242/dev.01600 15634696

[B32] Hafidh S. Fíla J. Honys D. (2016). Male gametophyte development and function in angiosperms: a general concept. *Plant Reproduc.* 29 31–51. 10.1007/s00497-015-0272-4 26728623

[B33] Hamza R. Pérez-Hedo M. Urbaneja A. Rambla J. L. Granell A. Gaddour K. (2018). Expression of two barley proteinase inhibitors in tomato promotes endogenous defensive response and enhances resistance to *Tuta absoluta*. *BMC Plant Biol.* 18:24. 10.1186/s12870-018-1240-6 29370757 PMC5785808

[B34] Hartley R. W. (1988). *Barnase* and *barstar*: expression of its cloned inhibitor permits expression of a cloned ribonuclease. *J. Mol. Biol.* 202 913–915. 3050134 10.1016/0022-2836(88)90568-2

[B35] Haywood V. Kragler F. Lucas W. J. (2002). Plasmodesmata: pathways for protein and ribonucleoprotein signaling. *Plant Cell* 14 S303–S325. 12045285 10.1105/tpc.000778 PMC151263

[B36] Heinlein M. (2002). Plasmodesmata: dynamic regulation and role in macromolecular cell-to-cell signaling. *Curr. Opin. Plant Biol.* 5 543–552. 10.1016/s1369-5266(02)00295-9 12393018

[B37] Heinlein M. Epel B. L. (2004). Macromolecular transport and signaling through plasmodesmata. *Int. Rev. Cytol.* 235 93–164. 15219782 10.1016/S0074-7696(04)35003-5

[B38] Higgins T. J. V. Beach L. R. Spencer D. Chandler P. M. Randall P. J. Blagrove R. J. (1987). cDNA and protein sequence of a major pea seed albumin (PA2: Mr 26.000). *Plant Mol. Biol.* 8 37–45. 10.1007/bf00016432 24302522

[B39] Higo K. Ugawa Y. Iwamoto M. Korenaga T. (1999). Plant cis-acting regulatory DNA elements (PLACE) database: 1999. *Nucleic Acids Res.* 27 297–300. 10.1093/nar/27.1.297 9847208 PMC148163

[B40] Ho A. Dowdy S. F. (2002). Regulation of G1 cell-cycle progression by oncogenes and tumor suppressor genes. *Curr. Opin. Genet. Dev.* 12 47–52. 10.1016/s0959-437x(01)00263-5 11790554

[B41] Hu L. Liang W. Yin C. Cui X. Zong J. Wang X. (2011). Rice MADS3 regulates ROS homeostasis during late anther development. *Plant Cell* 23 515–533. 21297036 10.1105/tpc.110.074369 PMC3077785

[B42] Huang H. Ullah F. Zhou D.-X. Yi M. Zhao Y. (2019). Mechanisms of ROS regulation of plant development and stress responses. *Front. Plant Sci.* 10:800. 10.3389/fpls.2019.00800 31293607 PMC6603150

[B43] Jacobowitz J. R. Doyle W. C. Weng J. K. (2019). PRX9 and PRX40 are extensin peroxidases essential for maintaining tapetum and microspore cell wall integrity during Arabidopsis anther development. *Plant Cell* 31 848–861. 10.1105/tpc.18.00907 30886127 PMC6501601

[B44] Jeney V. Balla J. Yachie A. Varga Z. Vercellotti G. M. Eaton J. W. (2002). Pro-oxidant and cytotoxic effects of circulating heme. *Blood* 100 879–887. 10.1182/blood.v100.3.879 12130498

[B45] Jorgensen P. Tyers M. (2004). How cells coordinate growth and division. *Curr. Biol.* 14 R1014–R1027. 15589139 10.1016/j.cub.2004.11.027

[B46] Kawanabe T. Ariizumi T. Kawai-Yamada M. Uchimiya H. Toriyama K. (2006). Abolition of the tapetum suicide program ruins microsporogenesis. *Plant Cell Physiol.* 47 784–787. 10.1093/pcp/pcj039 16565524

[B47] Ko S. S. Li M. J. Lin Y. J. Hsing H. X. Yang T. T. Chen T. K. (2017). Tightly controlled expression of bHLH142 is essential for timely tapetal programmed cell death and pollen development in rice. *Front. Plant Sci.* 8:1258. 10.3389/fpls.2017.01258 28769961 PMC5513933

[B48] Kumar S. Bandyopadhyay U. (2005). Free heme toxicity and its detoxification systems in human. *Toxicol. Lett.* 157 175–188. 15917143 10.1016/j.toxlet.2005.03.004

[B49] Lei X. Liu B. (2020). Tapetum-dependent male meiosis progression in plants: increasing evidence emerges. *Front. Plant Sci.* 10:1667. 10.3389/fpls.2019.01667 32010157 PMC6979054

[B50] Liu Z. Shi X. Li S. Hu G. Zhang L. Song X. (2018). Tapetal-delayed programmed cell death (PCD) and oxidative stress-induced male sterility of Aegilops uniaristata cytoplasm in wheat. *Int. J. Mol. Sci.* 19:1708. 10.3390/ijms19061708 29890696 PMC6032135

[B51] Livak K. J. Schmittgen T. D. (2001). Analysis of relative gene expression data using real-time quantitative PCR and the 2- ΔΔCT method. *Methods* 25 402–408. 10.1006/meth.2001.1262 11846609

[B52] Lodde V. Morandini P. Costa A. Murgia I. Ezquer I. (2021). cROStalk for life: uncovering ROS signaling in plants and animal systems, from gametogenesis to early embryonic development. *Genes* 12:525. 10.3390/genes12040525 33916807 PMC8067062

[B53] Luo D. Xu H. Liu Z. Guo J. Li H. Chen L. (2013). A detrimental mitochondrial-nuclear interaction causes cytoplasmic male sterility in rice. *Nat. Genet.* 45 573–577. 10.1038/ng.2570 23502780

[B54] Mariani C. De Beuckeleer M. Truettner J. Leemans J. Goldberg R. B. (1990). Induction of male sterility in plants by a chimaeric ribonuclease gene. *Nature* 347 737–741. 10.1038/347737a0

[B55] Mhamdi A. van Breusegem F. (2018). Reactive oxygen species in plant development. *Development* 145:dev164376. 10.1242/dev.164376 30093413

[B56] Miller G. A. D. Suzuki N. Ciftci-Yilmaz S. U. L. T. A. N. Mittler R. O. N. (2010). Reactive oxygen species homeostasis and signalling during drought and salinity stresses. *Plant Cell Environ.* 33 453–467. 19712065 10.1111/j.1365-3040.2009.02041.x

[B57] Mittler R. (2017). ROS are good. *Trends Plant Sci.* 22 11–19. 10.1016/j.tplants.2016.08.002 27666517

[B58] Mondol P. C. Xu D. Duan L. Shi J. Wang C. Chen X. (2020). Defective Pollen Wall 3 (DPW3), a novel alpha integrin-like protein, is required for pollen wall formation in rice. *New Phytol.* 225 807–822. 10.1111/nph.16161 31486533

[B59] Murmu J. Bush M. J. DeLong C. Li S. Xu M. Khan M. (2010). Arabidopsis basic leucine-zipper transcription factors TGA9 and TGA10 interact with floral glutaredoxins ROXY1 and ROXY2 and are redundantly required for anther development. *Plant Physiol.* 154 1492–1504. 10.1104/pp.110.159111 20805327 PMC2971623

[B60] Oh S. J. Song S. I Kim Y. S. Jang H. J. Kim S. Y. Kim M. (2005). Arabidopsis CBF3/DREB1A and ABF3 in transgenic rice increased tolerance to abiotic stress without stunting growth. *Plant Physiol.* 138 341–351. 10.1104/pp.104.059147 15834008 PMC1104188

[B61] Paoli M. Anderson B. F. Baker H. M. Morgan W. T. Smith A. Baker E. N. (1999). Crystal structure of hemopexin reveals a novel high-affinity heme site formed between two β-propeller domains. *Nat. Struc. Biol.* 6 926–931. 10.1038/13294 10504726

[B62] Parish R. W. Li S. F. (2010). Death of a tapetum: a programme of developmental altruism. *Plant Sci.* 178 73–89. 10.1016/j.plantsci.2009.11.001

[B63] Pettersen E. F. Goddard T. D. Huang C. C. Couch G. S. Greenblatt D. M. Meng E. C. (2004). UCSF Chimera-a visualization system for exploratory research and analysis. *J. Comput. Chem.* 25 1605–1612. 10.1002/jcc.20084 15264254

[B64] Pinkham J. L. Wang Z. Alsina J. (1997). Heme regulates SOD2 transcription by activation and repression in *Saccharomyces cerevisiae*. *Curr. Genet.* 31 281–291. 10.1007/s002940050207 9108135

[B65] Radice S. Ontivero M. Giordani E. Bellini E. (2008). Anatomical differences on development of fertile and sterile pollen grains of *Prunus salicina* Lindl. *Plant Syst. Evol.* 273 63–69. 10.1007/s00606-008-0011-5

[B66] Reichheld J. P. Vernoux T. Lardon F. Van Montagu M. Inzé D. (1999). Specific checkpoints regulate plant cell cycle progression in response to oxidative stress. *Plant J.* 17 647–656.

[B67] Riechmann J. L. Meyerowitz E. M. (1997). MADS-domain proteins in plant development. *Biol. Chem.* 378 1079–1102. 9372178

[B68] Robinson G. H. J. Domoney C. (2021). Perspectives on the genetic improvement of health- and nutrition-related traits in pea. *Plant Physiol. Biochem.* 158 353–362. 10.1016/j.plaphy.2020.11.020 33250319 PMC7801860

[B69] Rogers H. J. Bate N. Combe J. Sullivan J. Sweetman J. Swan C. (2001). Functional analysis of cis-regulatory elements within the promoter of the tobacco late pollen gene *g10*. *Plant Mol. Biol.* 45 577–585. 11414616 10.1023/a:1010695226241

[B70] Roque E. Gómez M. D. Ellul P. Wallbraun M. Madueño F. Beltrán J. P. (2007). The PsEND1 promoter: a novel tool to produce genetically engineered male-sterile plants by early anther ablation. *Plant Cell Rep.* 26 313–325. 10.1007/s00299-006-0237-z 17016735

[B71] Roque E. Hamza R. Gómez-Mena C. Beltrán J. P. Cañas L. A. (2019). Engineered male sterility by early anther ablation using the anther-specific promoter PsEND1. *Front. Plant Sci.* 10:819. 10.3389/fpls.2019.00819 31293612 PMC6603094

[B72] Sager R. Lee J. Y. (2014). Plasmodesmata in integrated cell signalling: insights from development and environmental signals and stresses. *J. Exper. Bot.* 65 6337–6358. 10.1093/jxb/eru365 25262225 PMC4303807

[B73] Sager R. E. Lee J. Y. (2018). Plasmodesmata at a glance. *J. Cell Sci.* 131:jcs209346. 10.1242/jcs.209346 29880547

[B74] Sanders P. M. Bui A. Q. Weterings K. McIntire K. N. Hsu Y. C. Lee P. Y. (1999). Anther developmental defects in *Arabidopsis thaliana* male-sterile mutants. *Sex. Plant Reproduc.* 11 297–322. 10.1007/s004970050158

[B75] Sankaranarayanan S. Ju Y. Kessler S. A. (2020). Reactive oxygen species as mediators of gametophyte development and double fertilization in flowering plants. *Front. Plant Sci.* 11:1199. 10.3389/fpls.2020.01199 32849744 PMC7419745

[B76] Schippers J. H. Foyer C. H. van Dongen J. T. (2016). Redox regulation in shoot growth, SAM maintenance and flowering. *Curr. Opin. Plant Biol.* 29 121–128. 10.1016/j.pbi.2015.11.009 26799134

[B77] Shore P. Sharrocks A. D. (1995). The MADS-box family of transcription factors. *Eur. J. Biochem.* 229 1–13. 7744019 10.1111/j.1432-1033.1995.tb20430.x

[B78] Shukla P. Gautam R. Singh N. K. Ahmed I. Kirti P. B. (2019). A proteomic study of cysteine protease induced cell death in anthers of male sterile tobacco transgenic plants. *Physiol. Mol. Biol. Plants* 25 1073–1082. 10.1007/s12298-019-00642-y 31402825 PMC6656835

[B79] Stahl Y. Simon R. (2013). Gated communities: apoplastic and symplastic signals converge at plasmodesmata to control cell fates. *J. Exper. Bot.* 64 5237–5241. 10.1093/jxb/ert245 23975796

[B80] Steer M. W. (1977). Differentiation of the tapetum in Avena. I. The cell surface. *J. Cell Sci.* 25 125–138. 10.1242/jcs.25.1.125 893555

[B81] Sun L. Xiang X. Yang Z. Yu P. Wen X. Wang H. (2018). *OsGPAT3* plays a critical role in anther wall programmed cell death and pollen development in rice. *Int. J. Mol. Sci.* 19:4017. 10.3390/ijms19124017 30545137 PMC6321289

[B82] Tang W. Perry S. E. (2003). Binding site selection for the plant MADS-domain protein AGL15: an *in vitro* and *in vivo* study. *J. Biol. Chem.* 278 28154–28159. 10.1074/jbc.m212976200 12743119

[B83] Tolosano E. Fagoonee S. Morello N. Vinchi F. Fiorito V. (2010). Heme-scavenging and the other facets of hemopexin. *Antioxid. Redox Signal.* 12 305–320. 10.1089/ars.2009.2787 19650691

[B84] Vercellotti G. M. Balla G. Balla J. Nath K. Eaton J. W. Jacob H. S. (1994). Heme and the vasculature: an oxidative hazard that induces antioxidant defenses in the endothelium. *Artif. Cells Blood Substit. Immobil. Biotechnol.* 22 207–213. 10.3109/10731199409117415 8087243

[B85] Vigeoles H. Chinoy C. Zuther E. Blessington B. Geigenberger P. Domoney C. (2008). Combined metabolomic and genetic approaches reveal a link between the polyamine pathway and albumin 2 in developing pea seeds. *Plant Physiol.* 146 74–82. 18024559 10.1104/pp.107.111369 PMC2230549

[B86] Wilson Z. A. Zhang D. B. (2009). From Arabidopsis to rice: pathways in pollen development. *J. Exper. Bot.* 60 1479–1492. 10.1093/jxb/erp095 19321648

[B87] Wu X. Weigel D. Wigge P. A. (2002). Signaling in plants by intercellular RNA and protein movement. *Genes Dev.* 16 151–158. 10.1101/gad.952002 11799058

[B88] Xie H. T. Wan Z. Y. Li S. Zhang Y. (2014). Spatiotemporal production of reactive oxygen species by NADPH oxidase is critical for tapetal programmed cell death and pollen development in *Arabidopsis*. *Plant Cell* 26 2007–2023. 10.1105/tpc.114.125427 24808050 PMC4079365

[B89] Xu D. Qu S. Tucker M. R. Zhang D. Liang W. Shi J. (2019). *Ostkpr1* functions in anther cuticle development and pollen wall formation in rice. *BMC Plant Biol.* 19:104. 10.1186/s12870-019-1711-4 30885140 PMC6421701

[B90] Yan M. Y. Xie D. L. Cao J. J. Xia X. J. Shi K. Zhou Y. H. (2020). Brassinosteroid-mediated reactive oxygen species are essential for tapetum degradation and pollen fertility in tomato. *Plant J.* 102 931–947. 10.1111/tpj.14672 31908046

[B91] Yanagisawa S. (2000). *Dof1* and *Dof2* transcription factors are associated with expression of multiple genes involved in carbon metabolism in maize. *Plant J.* 21 281–288. 10.1046/j.1365-313x.2000.00685.x 10758479

[B92] Yanagisawa S. Schmidt R. J. (1999). Diversity and similarity among recognition sequences of Dof transcription factors. *Plant J.* 17 209–214. 10074718 10.1046/j.1365-313x.1999.00363.x

[B93] Yi J. Moon S. Lee Y. S. Zhu L. Liang W. Zhang D. (2016). Defective tapetum cell death 1 (DTC1) regulates ROS levels by binding to metallothionein during tapetum degeneration. *Plant Physiol.* 170 1611–1623. 10.1104/pp.15.01561 26697896 PMC4775127

[B94] Zafra A. Rodríguez-García M. I. de Dios Alché J. (2010). Cellular localization of ROS and NO in olive reproductive tissues during flower development. *BMC Plant Biol.* 10:36. 10.1186/1471-2229-10-36 20181244 PMC2838403

[B95] Zhang X. Henriques R. Lin S. S. Niu Q. W. Chua N. H. (2006). Agrobacterium-mediated transformation of *Arabidopsis thaliana* using the floral dip method. *Nat. Protocols* 1 641–646. 17406292 10.1038/nprot.2006.97

[B96] Zheng S. Li J. Ma L. Wang H. Zhou H. Ni E. (2019). *OsAGO2* controls ROS production and the initiation of tapetal PCD by epigenetically regulating *OsHXK1* expression in rice anthers. *Proc. Natl. Acad. Sci.* 116 7549–7558. 30902896 10.1073/pnas.1817675116 PMC6462063

[B97] Zheng X. He L. Liu Y. Mao Y. Wang C. Zhao B. (2020). A study of male fertility control in *Medicago truncatula* uncovers an evolutionarily conserved recruitment of two tapetal bHLH subfamilies in plant sexual reproduction. *New Phytol.* 228 1115–1133. 32594537 10.1111/nph.16770

[B98] Zhu J. Lou Y. Xu X. Yang Z. N. (2011). A genetic pathway for tapetum development and function in Arabidopsis. *J. Integr. Plant Biol.* 53 892–900. 21957980 10.1111/j.1744-7909.2011.01078.x

