# Supplementary material for: PsEND1 Is a Key Player in Pea Pollen Development Through the Modulation of Redox Homeostasis
Source: Front Plant Sci. 2021 Oct 29;12:765277. doi: 10.3389/fpls.2021.765277 (PMC8586548; doi:10.3389/fpls.2021.765277)
Supplement: Supplementary file 1 [file Table_1.DOCX]

**Table S1. Primers used in this work.**

| **Primer** | **Sequence 5’-3’** |
| --- | --- |
| END1mutF | CGTCATTTATAGAGCCATTTTATGAAACACATATTTCATGTACACGTG |
| END1mutR | CACGTGTACATGAAATATGTGTTTCATAAAATGGCTCTATAAAATGACG |
| VIGSEND1F | ATGACAAAACCAGGTTAC |
| VIGSEND1R | CCCAGTGCTAATGTTCTTG |
| qEND1VIGSF | CTGTGCCTTTGACACCGATAA |
| qEND1VIGSR | TCTTCGGACCCGAGATGATT |
| qPsEF1F | GATGGATGCTACCACCCCTAAG |
| qPsEF1R | GAGATGGGAACGAAGGAATT |
| PsEND1ATG | ATGACAAAACCAGGTTACATTAATG |
| PsEND1STOP | CATTTAACTTTTCTGAGGTATTATAC |
