# Supplementary material for: PsEND1 Is a Key Player in Pea Pollen Development Through the Modulation of Redox Homeostasis
Source: Front Plant Sci. 2021 Oct 29;12:765277. doi: 10.3389/fpls.2021.765277 (PMC8586548; doi:10.3389/fpls.2021.765277)
Supplement: Supplementary file 2 [file Table_2.docx]

**Factor or Site Name Loc.(Str.) Signal Sequence SITE #**

**Table S2.** **Regulatory sequences found in the fragment -986/-6 of the *PsEND1* promoter.** Regulatory sequences frequently found in anthers are highlighted in blue.

____________________________________________________________________________

EBOXBNNAPA 7 (-) CANNTG [S000144](https://www.dna.affrc.go.jp/PLACE/?action=newPlaceSite&site=S000144)

MYCCONSENSUSAT 7 (-) CANNTG [S000407](https://www.dna.affrc.go.jp/PLACE/?action=newPlaceSite&site=S000407)

EBOXBNNAPA 7 (+) CANNTG [S000144](https://www.dna.affrc.go.jp/PLACE/?action=newPlaceSite&site=S000144)

MYCCONSENSUSAT 7 (+) CANNTG [S000407](https://www.dna.affrc.go.jp/PLACE/?action=newPlaceSite&site=S000407)

ARR1AT 13 (-) NGATT [S000454](https://www.dna.affrc.go.jp/PLACE/?action=newPlaceSite&site=S000454)

NONAMERMOTIFTAH3H4 16 (+) CATCCAACG [S000071](https://www.dna.affrc.go.jp/PLACE/?action=newPlaceSite&site=S000071)

ARR1AT 23 (+) NGATT [S000454](https://www.dna.affrc.go.jp/PLACE/?action=newPlaceSite&site=S000454)

EBOXBNNAPA 29 (-) CANNTG [S000144](https://www.dna.affrc.go.jp/PLACE/?action=newPlaceSite&site=S000144)

MYCCONSENSUSAT 29 (-) CANNTG [S000407](https://www.dna.affrc.go.jp/PLACE/?action=newPlaceSite&site=S000407)

MYB2CONSENSUSAT 29 (-) YAACKG [S000409](https://www.dna.affrc.go.jp/PLACE/?action=newPlaceSite&site=S000409)

EBOXBNNAPA 29 (+) CANNTG [S000144](https://www.dna.affrc.go.jp/PLACE/?action=newPlaceSite&site=S000144)

MYBCORE 29 (+) CNGTTR [S000176](https://www.dna.affrc.go.jp/PLACE/?action=newPlaceSite&site=S000176)

MYCCONSENSUSAT 29 (+) CANNTG [S000407](https://www.dna.affrc.go.jp/PLACE/?action=newPlaceSite&site=S000407)

RAV1AAT 33 (-) CAACA [S000314](https://www.dna.affrc.go.jp/PLACE/?action=newPlaceSite&site=S000314)

CGACGOSAMY3 39 (-) CGACG [S000205](https://www.dna.affrc.go.jp/PLACE/?action=newPlaceSite&site=S000205)

CAATBOX1 46 (+) CAAT [S000028](https://www.dna.affrc.go.jp/PLACE/?action=newPlaceSite&site=S000028)

ARR1AT 65 (+) NGATT [S000454](https://www.dna.affrc.go.jp/PLACE/?action=newPlaceSite&site=S000454)

GATABOX 77 (+) GATA [S000039](https://www.dna.affrc.go.jp/PLACE/?action=newPlaceSite&site=S000039)

ROOTMOTIFTAPOX1 80 (+) ATATT [S000098](https://www.dna.affrc.go.jp/PLACE/?action=newPlaceSite&site=S000098)

ANAERO1CONSENSUS 91 (-) AAACAAA [S000477](https://www.dna.affrc.go.jp/PLACE/?action=newPlaceSite&site=S000477)

GT1CONSENSUS 116 (-) GRWAAW [S000198](https://www.dna.affrc.go.jp/PLACE/?action=newPlaceSite&site=S000198)

POLASIG3 122 (-) AATAAT [S000088](https://www.dna.affrc.go.jp/PLACE/?action=newPlaceSite&site=S000088)

-10PEHVPSBD 124 (+) TATTCT [S000392](https://www.dna.affrc.go.jp/PLACE/?action=newPlaceSite&site=S000392)

CACTFTPPCA1 144 (+) YACT [S000449](https://www.dna.affrc.go.jp/PLACE/?action=newPlaceSite&site=S000449)

-300ELEMENT 157 (+) TGHAAARK [S000122](https://www.dna.affrc.go.jp/PLACE/?action=newPlaceSite&site=S000122)

PYRIMIDINEBOXOSRAMY1A 160 (-) CCTTTT [S000259](https://www.dna.affrc.go.jp/PLACE/?action=newPlaceSite&site=S000259)

DOFCOREZM 161 (+) AAAG [S000265](https://www.dna.affrc.go.jp/PLACE/?action=newPlaceSite&site=S000265)

POLASIG1 166 (+) AATAAA [S000080](https://www.dna.affrc.go.jp/PLACE/?action=newPlaceSite&site=S000080)

TAAAGSTKST1 168 (+) TAAAG [S000387](https://www.dna.affrc.go.jp/PLACE/?action=newPlaceSite&site=S000387)

DOFCOREZM 169 (+) AAAG [S000265](https://www.dna.affrc.go.jp/PLACE/?action=newPlaceSite&site=S000265)

NODCON1GM 169 (+) AAAGAT [S000461](https://www.dna.affrc.go.jp/PLACE/?action=newPlaceSite&site=S000461)

OSE1ROOTNODULE 169 (+) AAAGAT [S000467](https://www.dna.affrc.go.jp/PLACE/?action=newPlaceSite&site=S000467)

GATABOX 172 (+) GATA [S000039](https://www.dna.affrc.go.jp/PLACE/?action=newPlaceSite&site=S000039)

CAATBOX1 176 (+) CAAT [S000028](https://www.dna.affrc.go.jp/PLACE/?action=newPlaceSite&site=S000028)

GTGANTG10 184 (-) GTGA [S000378](https://www.dna.affrc.go.jp/PLACE/?action=newPlaceSite&site=S000378)

CACTFTPPCA1 190 (+) YACT [S000449](https://www.dna.affrc.go.jp/PLACE/?action=newPlaceSite&site=S000449)

GTGANTG10 193 (-) GTGA [S000378](https://www.dna.affrc.go.jp/PLACE/?action=newPlaceSite&site=S000378)

EBOXBNNAPA 194 (-) CANNTG [S000144](https://www.dna.affrc.go.jp/PLACE/?action=newPlaceSite&site=S000144)

MYCCONSENSUSAT 194 (-) CANNTG [S000407](https://www.dna.affrc.go.jp/PLACE/?action=newPlaceSite&site=S000407)

EBOXBNNAPA 194 (+) CANNTG [S000144](https://www.dna.affrc.go.jp/PLACE/?action=newPlaceSite&site=S000144)

MYCCONSENSUSAT 194 (+) CANNTG [S000407](https://www.dna.affrc.go.jp/PLACE/?action=newPlaceSite&site=S000407)

CACTFTPPCA1 194 (+) YACT [S000449](https://www.dna.affrc.go.jp/PLACE/?action=newPlaceSite&site=S000449)

ROOTMOTIFTAPOX1 204 (-) ATATT [S000098](https://www.dna.affrc.go.jp/PLACE/?action=newPlaceSite&site=S000098)

POLLEN1LELAT52 225 (+) AGAAA [S000245](https://www.dna.affrc.go.jp/PLACE/?action=newPlaceSite&site=S000245)

CACTFTPPCA1 230 (+) YACT [S000449](https://www.dna.affrc.go.jp/PLACE/?action=newPlaceSite&site=S000449)

CCA1ATLHCB1 265 (+) AAMAATCT [S000149](https://www.dna.affrc.go.jp/PLACE/?action=newPlaceSite&site=S000149)

CAATBOX1 267 (+) CAAT [S000028](https://www.dna.affrc.go.jp/PLACE/?action=newPlaceSite&site=S000028)

ARR1AT 268 (-) NGATT [S000454](https://www.dna.affrc.go.jp/PLACE/?action=newPlaceSite&site=S000454)

ARR1AT 287 (-) NGATT [S000454](https://www.dna.affrc.go.jp/PLACE/?action=newPlaceSite&site=S000454)

WUSATAg 290 (-) TTAATGG [S000433](https://www.dna.affrc.go.jp/PLACE/?action=newPlaceSite&site=S000433)

CACTFTPPCA1 298 (+) YACT [S000449](https://www.dna.affrc.go.jp/PLACE/?action=newPlaceSite&site=S000449)

TBOXATGAPB 299 (+) ACTTTG [S000383](https://www.dna.affrc.go.jp/PLACE/?action=newPlaceSite&site=S000383)

DOFCOREZM 300 (-) AAAG [S000265](https://www.dna.affrc.go.jp/PLACE/?action=newPlaceSite&site=S000265)

GAREAT 315 (+) TAACAAR [S000439](https://www.dna.affrc.go.jp/PLACE/?action=newPlaceSite&site=S000439)

CACTFTPPCA1 332 (-) YACT [S000449](https://www.dna.affrc.go.jp/PLACE/?action=newPlaceSite&site=S000449)

ROOTMOTIFTAPOX1 341 (+) ATATT [S000098](https://www.dna.affrc.go.jp/PLACE/?action=newPlaceSite&site=S000098)

CPBCSPOR 342 (+) TATTAG [S000491](https://www.dna.affrc.go.jp/PLACE/?action=newPlaceSite&site=S000491)

NODCON2GM 349 (+) CTCTT [S000462](https://www.dna.affrc.go.jp/PLACE/?action=newPlaceSite&site=S000462)

OSE2ROOTNODULE 349 (+) CTCTT [S000468](https://www.dna.affrc.go.jp/PLACE/?action=newPlaceSite&site=S000468)

TATABOX5 356 (+) TTATTT [S000203](https://www.dna.affrc.go.jp/PLACE/?action=newPlaceSite&site=S000203)

SEF4MOTIFGM7S 358 (+) RTTTTTR [S000103](https://www.dna.affrc.go.jp/PLACE/?action=newPlaceSite&site=S000103)

ROOTMOTIFTAPOX1 364 (+) ATATT [S000098](https://www.dna.affrc.go.jp/PLACE/?action=newPlaceSite&site=S000098)

GT1CONSENSUS 366 (-) GRWAAW [S000198](https://www.dna.affrc.go.jp/PLACE/?action=newPlaceSite&site=S000198)

IBOXCORE 367 (-) GATAA [S000199](https://www.dna.affrc.go.jp/PLACE/?action=newPlaceSite&site=S000199)

GATABOX 368 (-) GATA [S000039](https://www.dna.affrc.go.jp/PLACE/?action=newPlaceSite&site=S000039)

DOFCOREZM 376 (-) AAAG [S000265](https://www.dna.affrc.go.jp/PLACE/?action=newPlaceSite&site=S000265)

POLLEN1LELAT52 378 (-) AGAAA [S000245](https://www.dna.affrc.go.jp/PLACE/?action=newPlaceSite&site=S000245)

GTGANTG10 394 (-) GTGA [S000378](https://www.dna.affrc.go.jp/PLACE/?action=newPlaceSite&site=S000378)

CACTFTPPCA1 395 (+) YACT [S000449](https://www.dna.affrc.go.jp/PLACE/?action=newPlaceSite&site=S000449)

CPBCSPOR 397 (-) TATTAG [S000491](https://www.dna.affrc.go.jp/PLACE/?action=newPlaceSite&site=S000491)

ROOTMOTIFTAPOX1 399 (-) ATATT [S000098](https://www.dna.affrc.go.jp/PLACE/?action=newPlaceSite&site=S000098)

GATABOX 404 (+) GATA [S000039](https://www.dna.affrc.go.jp/PLACE/?action=newPlaceSite&site=S000039)

ROOTMOTIFTAPOX1 405 (+) ATATT [S000098](https://www.dna.affrc.go.jp/PLACE/?action=newPlaceSite&site=S000098)

GT1CONSENSUS 407 (-) GRWAAW [S000198](https://www.dna.affrc.go.jp/PLACE/?action=newPlaceSite&site=S000198)

IBOXCORE 408 (-) GATAA [S000199](https://www.dna.affrc.go.jp/PLACE/?action=newPlaceSite&site=S000199)

GATABOX 409 (-) GATA [S000039](https://www.dna.affrc.go.jp/PLACE/?action=newPlaceSite&site=S000039)

TATABOX5 427 (+) TTATTT [S000203](https://www.dna.affrc.go.jp/PLACE/?action=newPlaceSite&site=S000203)

TATABOX2 429 (-) TATAAAT [S000109](https://www.dna.affrc.go.jp/PLACE/?action=newPlaceSite&site=S000109)

MARARS 429 (+) WTTTATRTTTW [S000064](https://www.dna.affrc.go.jp/PLACE/?action=newPlaceSite&site=S000064)

SEF1MOTIF 433 (+) ATATTTAWW [S000006](https://www.dna.affrc.go.jp/PLACE/?action=newPlaceSite&site=S000006)

ROOTMOTIFTAPOX1 433 (+) ATATT [S000098](https://www.dna.affrc.go.jp/PLACE/?action=newPlaceSite&site=S000098)

POLASIG1 436 (-) AATAAA [S000080](https://www.dna.affrc.go.jp/PLACE/?action=newPlaceSite&site=S000080)

CPBCSPOR 438 (+) TATTAG [S000491](https://www.dna.affrc.go.jp/PLACE/?action=newPlaceSite&site=S000491)

POLLEN1LELAT52 442 (+) AGAAA [S000245](https://www.dna.affrc.go.jp/PLACE/?action=newPlaceSite&site=S000245)

GT1CONSENSUS 443 (+) GRWAAW [S000198](https://www.dna.affrc.go.jp/PLACE/?action=newPlaceSite&site=S000198)

GT1GMSCAM4 443 (+) GAAAAA [S000453](https://www.dna.affrc.go.jp/PLACE/?action=newPlaceSite&site=S000453)

DOFCOREZM 446 (+) AAAG [S000265](https://www.dna.affrc.go.jp/PLACE/?action=newPlaceSite&site=S000265)

NODCON2GM 447 (-) CTCTT [S000462](https://www.dna.affrc.go.jp/PLACE/?action=newPlaceSite&site=S000462)

OSE2ROOTNODULE 447 (-) CTCTT [S000468](https://www.dna.affrc.go.jp/PLACE/?action=newPlaceSite&site=S000468)

EECCRCAH1 449 (+) GANTTNC [S000494](https://www.dna.affrc.go.jp/PLACE/?action=newPlaceSite&site=S000494)

INRNTPSADB 453 (+) YTCANTYY [S000395](https://www.dna.affrc.go.jp/PLACE/?action=newPlaceSite&site=S000395)

SEF4MOTIFGM7S 462 (-) RTTTTTR [S000103](https://www.dna.affrc.go.jp/PLACE/?action=newPlaceSite&site=S000103)

ROOTMOTIFTAPOX1 466 (-) ATATT [S000098](https://www.dna.affrc.go.jp/PLACE/?action=newPlaceSite&site=S000098)

ROOTMOTIFTAPOX1 469 (+) ATATT [S000098](https://www.dna.affrc.go.jp/PLACE/?action=newPlaceSite&site=S000098)

GT1CONSENSUS 471 (-) GRWAAW [S000198](https://www.dna.affrc.go.jp/PLACE/?action=newPlaceSite&site=S000198)

-300ELEMENT 480 (-) TGHAAARK [S000122](https://www.dna.affrc.go.jp/PLACE/?action=newPlaceSite&site=S000122)

GT1CONSENSUS 481 (-) GRWAAW [S000198](https://www.dna.affrc.go.jp/PLACE/?action=newPlaceSite&site=S000198)

GT1GMSCAM4 481 (-) GAAAAA [S000453](https://www.dna.affrc.go.jp/PLACE/?action=newPlaceSite&site=S000453)

TATABOX5 490 (-) TTATTT [S000203](https://www.dna.affrc.go.jp/PLACE/?action=newPlaceSite&site=S000203)

POLASIG1 491 (+) AATAAA [S000080](https://www.dna.affrc.go.jp/PLACE/?action=newPlaceSite&site=S000080)

SEF1MOTIF 499 (-) ATATTTAWW [S000006](https://www.dna.affrc.go.jp/PLACE/?action=newPlaceSite&site=S000006)

TATABOXOSPAL 500 (-) TATTTAA [S000400](https://www.dna.affrc.go.jp/PLACE/?action=newPlaceSite&site=S000400)

ROOTMOTIFTAPOX1 503 (-) ATATT [S000098](https://www.dna.affrc.go.jp/PLACE/?action=newPlaceSite&site=S000098)

LECPLEACS2 506 (-) TAAAATAT [S000465](https://www.dna.affrc.go.jp/PLACE/?action=newPlaceSite&site=S000465)

ROOTMOTIFTAPOX1 506 (+) ATATT [S000098](https://www.dna.affrc.go.jp/PLACE/?action=newPlaceSite&site=S000098)

POLASIG3 521 (-) AATAAT [S000088](https://www.dna.affrc.go.jp/PLACE/?action=newPlaceSite&site=S000088)

TATABOX5 522 (+) TTATTT [S000203](https://www.dna.affrc.go.jp/PLACE/?action=newPlaceSite&site=S000203)

TATABOXOSPAL 523 (+) TATTTAA [S000400](https://www.dna.affrc.go.jp/PLACE/?action=newPlaceSite&site=S000400)

POLASIG3 528 (+) AATAAT [S000088](https://www.dna.affrc.go.jp/PLACE/?action=newPlaceSite&site=S000088)

POLASIG1 533 (-) AATAAA [S000080](https://www.dna.affrc.go.jp/PLACE/?action=newPlaceSite&site=S000080)

TATABOX5 534 (+) TTATTT [S000203](https://www.dna.affrc.go.jp/PLACE/?action=newPlaceSite&site=S000203)

MARARS 551 (-) WTTTATRTTTW [S000064](https://www.dna.affrc.go.jp/PLACE/?action=newPlaceSite&site=S000064)

ROOTMOTIFTAPOX1 553 (-) ATATT [S000098](https://www.dna.affrc.go.jp/PLACE/?action=newPlaceSite&site=S000098)

TATABOX2 555 (+) TATAAAT [S000109](https://www.dna.affrc.go.jp/PLACE/?action=newPlaceSite&site=S000109)

TATABOX5 558 (-) TTATTT [S000203](https://www.dna.affrc.go.jp/PLACE/?action=newPlaceSite&site=S000203)

POLASIG1 559 (+) AATAAA [S000080](https://www.dna.affrc.go.jp/PLACE/?action=newPlaceSite&site=S000080)

BIHD1OS 569 (+) TGTCA [S000498](https://www.dna.affrc.go.jp/PLACE/?action=newPlaceSite&site=S000498)

WRKY71OS 570 (-) TGAC [S000447](https://www.dna.affrc.go.jp/PLACE/?action=newPlaceSite&site=S000447)

ROOTMOTIFTAPOX1 577 (-) ATATT [S000098](https://www.dna.affrc.go.jp/PLACE/?action=newPlaceSite&site=S000098)

ROOTMOTIFTAPOX1 578 (+) ATATT [S000098](https://www.dna.affrc.go.jp/PLACE/?action=newPlaceSite&site=S000098)

TATABOXOSPAL 579 (+) TATTTAA [S000400](https://www.dna.affrc.go.jp/PLACE/?action=newPlaceSite&site=S000400)

GT1CORE 582 (-) GGTTAA [S000125](https://www.dna.affrc.go.jp/PLACE/?action=newPlaceSite&site=S000125)

PYRIMIDINEBOXOSRAMY1A 586 (+) CCTTTT [S000259](https://www.dna.affrc.go.jp/PLACE/?action=newPlaceSite&site=S000259)

DOFCOREZM 587 (-) AAAG [S000265](https://www.dna.affrc.go.jp/PLACE/?action=newPlaceSite&site=S000265)

CAATBOX1 594 (+) CAAT [S000028](https://www.dna.affrc.go.jp/PLACE/?action=newPlaceSite&site=S000028)

POLASIG1 595 (+) AATAAA [S000080](https://www.dna.affrc.go.jp/PLACE/?action=newPlaceSite&site=S000080)

SEF1MOTIF 603 (+) ATATTTAWW [S000006](https://www.dna.affrc.go.jp/PLACE/?action=newPlaceSite&site=S000006)

ROOTMOTIFTAPOX1 603 (+) ATATT [S000098](https://www.dna.affrc.go.jp/PLACE/?action=newPlaceSite&site=S000098)

POLASIG1 606 (-) AATAAA [S000080](https://www.dna.affrc.go.jp/PLACE/?action=newPlaceSite&site=S000080)

TATABOX5 607 (+) TTATTT [S000203](https://www.dna.affrc.go.jp/PLACE/?action=newPlaceSite&site=S000203)

TATABOXOSPAL 608 (+) TATTTAA [S000400](https://www.dna.affrc.go.jp/PLACE/?action=newPlaceSite&site=S000400)

POLASIG2 610 (-) AATTAAA [S000081](https://www.dna.affrc.go.jp/PLACE/?action=newPlaceSite&site=S000081)

CARGCW8GAT 620 (-) CWWWWWWWWG [S000431](https://www.dna.affrc.go.jp/PLACE/?action=newPlaceSite&site=S000431)

CPBCSPOR 620 (-) TATTAG [S000491](https://www.dna.affrc.go.jp/PLACE/?action=newPlaceSite&site=S000491)

CARGCW8GAT 620 (+) CWWWWWWWWG [S000431](https://www.dna.affrc.go.jp/PLACE/?action=newPlaceSite&site=S000431)

ROOTMOTIFTAPOX1 622 (-) ATATT [S000098](https://www.dna.affrc.go.jp/PLACE/?action=newPlaceSite&site=S000098)

MYBPZM 645 (+) CCWACC [S000179](https://www.dna.affrc.go.jp/PLACE/?action=newPlaceSite&site=S000179)

REALPHALGLHCB21 647 (+) AACCAA [S000362](https://www.dna.affrc.go.jp/PLACE/?action=newPlaceSite&site=S000362)

CCAATBOX1 649 (+) CCAAT [S000030](https://www.dna.affrc.go.jp/PLACE/?action=newPlaceSite&site=S000030)

CAATBOX1 650 (+) CAAT [S000028](https://www.dna.affrc.go.jp/PLACE/?action=newPlaceSite&site=S000028)

POLASIG2 651 (+) AATTAAA [S000081](https://www.dna.affrc.go.jp/PLACE/?action=newPlaceSite&site=S000081)

DOFCOREZM 657 (+) AAAG [S000265](https://www.dna.affrc.go.jp/PLACE/?action=newPlaceSite&site=S000265)

NODCON1GM 657 (+) AAAGAT [S000461](https://www.dna.affrc.go.jp/PLACE/?action=newPlaceSite&site=S000461)

OSE1ROOTNODULE 657 (+) AAAGAT [S000467](https://www.dna.affrc.go.jp/PLACE/?action=newPlaceSite&site=S000467)

EBOXBNNAPA 663 (-) CANNTG [S000144](https://www.dna.affrc.go.jp/PLACE/?action=newPlaceSite&site=S000144)

MYCCONSENSUSAT 663 (-) CANNTG [S000407](https://www.dna.affrc.go.jp/PLACE/?action=newPlaceSite&site=S000407)

EBOXBNNAPA 663 (+) CANNTG [S000144](https://www.dna.affrc.go.jp/PLACE/?action=newPlaceSite&site=S000144)

MYCCONSENSUSAT 663 (+) CANNTG [S000407](https://www.dna.affrc.go.jp/PLACE/?action=newPlaceSite&site=S000407)

GT1CONSENSUS 668 (+) GRWAAW [S000198](https://www.dna.affrc.go.jp/PLACE/?action=newPlaceSite&site=S000198)

PYRIMIDINEBOXOSRAMY1A 675 (+) CCTTTT [S000259](https://www.dna.affrc.go.jp/PLACE/?action=newPlaceSite&site=S000259)

DOFCOREZM 676 (-) AAAG [S000265](https://www.dna.affrc.go.jp/PLACE/?action=newPlaceSite&site=S000265)

POLASIG1 679 (-) AATAAA [S000080](https://www.dna.affrc.go.jp/PLACE/?action=newPlaceSite&site=S000080)

TATABOX5 680 (+) TTATTT [S000203](https://www.dna.affrc.go.jp/PLACE/?action=newPlaceSite&site=S000203)

SORLIP1AT 691 (-) GCCAC [S000482](https://www.dna.affrc.go.jp/PLACE/?action=newPlaceSite&site=S000482)

EBOXBNNAPA 701 (-) CANNTG [S000144](https://www.dna.affrc.go.jp/PLACE/?action=newPlaceSite&site=S000144)

MYBCORE 701 (-) CNGTTR [S000176](https://www.dna.affrc.go.jp/PLACE/?action=newPlaceSite&site=S000176)

MYCCONSENSUSAT 701 (-) CANNTG [S000407](https://www.dna.affrc.go.jp/PLACE/?action=newPlaceSite&site=S000407)

EBOXBNNAPA 701 (+) CANNTG [S000144](https://www.dna.affrc.go.jp/PLACE/?action=newPlaceSite&site=S000144)

MYCCONSENSUSAT 701 (+) CANNTG [S000407](https://www.dna.affrc.go.jp/PLACE/?action=newPlaceSite&site=S000407)

MYB2CONSENSUSAT 701 (+) YAACKG [S000409](https://www.dna.affrc.go.jp/PLACE/?action=newPlaceSite&site=S000409)

BIHD1OS 705 (+) TGTCA [S000498](https://www.dna.affrc.go.jp/PLACE/?action=newPlaceSite&site=S000498)

WRKY71OS 706 (-) TGAC [S000447](https://www.dna.affrc.go.jp/PLACE/?action=newPlaceSite&site=S000447)

PYRIMIDINEBOXOSRAMY1A 714 (-) CCTTTT [S000259](https://www.dna.affrc.go.jp/PLACE/?action=newPlaceSite&site=S000259)

DOFCOREZM 715 (+) AAAG [S000265](https://www.dna.affrc.go.jp/PLACE/?action=newPlaceSite&site=S000265)

SEF4MOTIFGM7S 721 (+) RTTTTTR [S000103](https://www.dna.affrc.go.jp/PLACE/?action=newPlaceSite&site=S000103)

CGACGOSAMY3 728 (+) CGACG [S000205](https://www.dna.affrc.go.jp/PLACE/?action=newPlaceSite&site=S000205)

GATABOX 732 (+) GATA [S000039](https://www.dna.affrc.go.jp/PLACE/?action=newPlaceSite&site=S000039)

TATABOX2 734 (+) TATAAAT [S000109](https://www.dna.affrc.go.jp/PLACE/?action=newPlaceSite&site=S000109)

MYB1AT 743 (+) WAACCA [S000408](https://www.dna.affrc.go.jp/PLACE/?action=newPlaceSite&site=S000408)

CARGCW8GAT 750 (-) CWWWWWWWWG [S000431](https://www.dna.affrc.go.jp/PLACE/?action=newPlaceSite&site=S000431)

CARGCW8GAT 750 (+) CWWWWWWWWG [S000431](https://www.dna.affrc.go.jp/PLACE/?action=newPlaceSite&site=S000431)

INRNTPSADB 754 (-) YTCANTYY [S000395](https://www.dna.affrc.go.jp/PLACE/?action=newPlaceSite&site=S000395)

CAATBOX1 756 (-) CAAT [S000028](https://www.dna.affrc.go.jp/PLACE/?action=newPlaceSite&site=S000028)

MYB2AT 763 (-) TAACTG [S000177](https://www.dna.affrc.go.jp/PLACE/?action=newPlaceSite&site=S000177)

MYB2CONSENSUSAT 763 (-) YAACKG [S000409](https://www.dna.affrc.go.jp/PLACE/?action=newPlaceSite&site=S000409)

MYBCORE 763 (+) CNGTTR [S000176](https://www.dna.affrc.go.jp/PLACE/?action=newPlaceSite&site=S000176)

REALPHALGLHCB21 773 (+) AACCAA [S000362](https://www.dna.affrc.go.jp/PLACE/?action=newPlaceSite&site=S000362)

S1FBOXSORPS1L21 783 (+) ATGGTA [S000223](https://www.dna.affrc.go.jp/PLACE/?action=newPlaceSite&site=S000223)

TATCCAYMOTIFOSRAMY3D 790 (-) TATCCAY [S000256](https://www.dna.affrc.go.jp/PLACE/?action=newPlaceSite&site=S000256)

TATCCACHVAL21 790 (-) TATCCAC [S000416](https://www.dna.affrc.go.jp/PLACE/?action=newPlaceSite&site=S000416)

TATCCAOSAMY 791 (-) TATCCA [S000403](https://www.dna.affrc.go.jp/PLACE/?action=newPlaceSite&site=S000403)

MYBST1 792 (+) GGATA [S000180](https://www.dna.affrc.go.jp/PLACE/?action=newPlaceSite&site=S000180)

GATABOX 793 (+) GATA [S000039](https://www.dna.affrc.go.jp/PLACE/?action=newPlaceSite&site=S000039)

ROOTMOTIFTAPOX1 796 (+) ATATT [S000098](https://www.dna.affrc.go.jp/PLACE/?action=newPlaceSite&site=S000098)

GT1CONSENSUS 799 (-) GRWAAW [S000198](https://www.dna.affrc.go.jp/PLACE/?action=newPlaceSite&site=S000198)

IBOXCORE 800 (-) GATAA [S000199](https://www.dna.affrc.go.jp/PLACE/?action=newPlaceSite&site=S000199)

GATABOX 801 (-) GATA [S000039](https://www.dna.affrc.go.jp/PLACE/?action=newPlaceSite&site=S000039)

NODCON1GM 802 (-) AAAGAT [S000461](https://www.dna.affrc.go.jp/PLACE/?action=newPlaceSite&site=S000461)

OSE1ROOTNODULE 802 (-) AAAGAT [S000467](https://www.dna.affrc.go.jp/PLACE/?action=newPlaceSite&site=S000467)

DOFCOREZM 804 (-) AAAG [S000265](https://www.dna.affrc.go.jp/PLACE/?action=newPlaceSite&site=S000265)

GATABOX 816 (-) GATA [S000039](https://www.dna.affrc.go.jp/PLACE/?action=newPlaceSite&site=S000039)

ARR1AT 820 (+) NGATT [S000454](https://www.dna.affrc.go.jp/PLACE/?action=newPlaceSite&site=S000454)

ARR1AT 827 (-) NGATT [S000454](https://www.dna.affrc.go.jp/PLACE/?action=newPlaceSite&site=S000454)

-300ELEMENT 841 (+) TGHAAARK [S000122](https://www.dna.affrc.go.jp/PLACE/?action=newPlaceSite&site=S000122)

GT1CONSENSUS 842 (+) GRWAAW [S000198](https://www.dna.affrc.go.jp/PLACE/?action=newPlaceSite&site=S000198)

GT1GMSCAM4 842 (+) GAAAAA [S000453](https://www.dna.affrc.go.jp/PLACE/?action=newPlaceSite&site=S000453)

GATABOX 853 (-) GATA [S000039](https://www.dna.affrc.go.jp/PLACE/?action=newPlaceSite&site=S000039)

NTBBF1ARROLB 859 (+) ACTTTA [S000273](https://www.dna.affrc.go.jp/PLACE/?action=newPlaceSite&site=S000273)

DOFCOREZM 860 (-) AAAG [S000265](https://www.dna.affrc.go.jp/PLACE/?action=newPlaceSite&site=S000265)

TAAAGSTKST1 860 (-) TAAAG [S000387](https://www.dna.affrc.go.jp/PLACE/?action=newPlaceSite&site=S000387)

TATABOXOSPAL 862 (-) TATTTAA [S000400](https://www.dna.affrc.go.jp/PLACE/?action=newPlaceSite&site=S000400)

TATABOX5 864 (-) TTATTT [S000203](https://www.dna.affrc.go.jp/PLACE/?action=newPlaceSite&site=S000203)

TGACGTVMAMY 872 (-) TGACGT [S000377](https://www.dna.affrc.go.jp/PLACE/?action=newPlaceSite&site=S000377)

ACGTATERD1 872 (-) ACGT [S000415](https://www.dna.affrc.go.jp/PLACE/?action=newPlaceSite&site=S000415)

HEXMOTIFTAH3H4 872 (+) ACGTCA [S000053](https://www.dna.affrc.go.jp/PLACE/?action=newPlaceSite&site=S000053)

ACGTATERD1 872 (+) ACGT [S000415](https://www.dna.affrc.go.jp/PLACE/?action=newPlaceSite&site=S000415)

ASF1MOTIFCAMV 873 (-) TGACG [S000024](https://www.dna.affrc.go.jp/PLACE/?action=newPlaceSite&site=S000024)

WRKY71OS 874 (-) TGAC [S000447](https://www.dna.affrc.go.jp/PLACE/?action=newPlaceSite&site=S000447)

TATABOX2 877 (-) TATAAAT [S000109](https://www.dna.affrc.go.jp/PLACE/?action=newPlaceSite&site=S000109)

ROOTMOTIFTAPOX1 903 (+) ATATT [S000098](https://www.dna.affrc.go.jp/PLACE/?action=newPlaceSite&site=S000098)

CURECORECR 912 (-) GTAC [S000493](https://www.dna.affrc.go.jp/PLACE/?action=newPlaceSite&site=S000493)

CURECORECR 912 (+) GTAC [S000493](https://www.dna.affrc.go.jp/PLACE/?action=newPlaceSite&site=S000493)

ABRERATCAL 914 (-) MACGYGB [S000507](https://www.dna.affrc.go.jp/PLACE/?action=newPlaceSite&site=S000507)

DPBFCOREDCDC3 914 (+) ACACNNG [S000292](https://www.dna.affrc.go.jp/PLACE/?action=newPlaceSite&site=S000292)

CACGTGMOTIF 915 (-) CACGTG [S000042](https://www.dna.affrc.go.jp/PLACE/?action=newPlaceSite&site=S000042)

EBOXBNNAPA 915 (-) CANNTG [S000144](https://www.dna.affrc.go.jp/PLACE/?action=newPlaceSite&site=S000144)

MYCCONSENSUSAT 915 (-) CANNTG [S000407](https://www.dna.affrc.go.jp/PLACE/?action=newPlaceSite&site=S000407)

ABRELATERD1 915 (-) ACGTG [S000414](https://www.dna.affrc.go.jp/PLACE/?action=newPlaceSite&site=S000414)

CACGTGMOTIF 915 (+) CACGTG [S000042](https://www.dna.affrc.go.jp/PLACE/?action=newPlaceSite&site=S000042)

EBOXBNNAPA 915 (+) CANNTG [S000144](https://www.dna.affrc.go.jp/PLACE/?action=newPlaceSite&site=S000144)

MYCCONSENSUSAT 915 (+) CANNTG [S000407](https://www.dna.affrc.go.jp/PLACE/?action=newPlaceSite&site=S000407)

ACGTATERD1 916 (-) ACGT [S000415](https://www.dna.affrc.go.jp/PLACE/?action=newPlaceSite&site=S000415)

RHERPATEXPA7 916 (-) KCACGW [S000512](https://www.dna.affrc.go.jp/PLACE/?action=newPlaceSite&site=S000512)

ABRELATERD1 916 (+) ACGTG [S000414](https://www.dna.affrc.go.jp/PLACE/?action=newPlaceSite&site=S000414)

ACGTATERD1 916 (+) ACGT [S000415](https://www.dna.affrc.go.jp/PLACE/?action=newPlaceSite&site=S000415)

GTGANTG10 918 (+) GTGA [S000378](https://www.dna.affrc.go.jp/PLACE/?action=newPlaceSite&site=S000378)

GT1CONSENSUS 930 (-) GRWAAW [S000198](https://www.dna.affrc.go.jp/PLACE/?action=newPlaceSite&site=S000198)

CCAATBOX1 934 (+) CCAAT [S000030](https://www.dna.affrc.go.jp/PLACE/?action=newPlaceSite&site=S000030)

CAATBOX1 935 (+) CAAT [S000028](https://www.dna.affrc.go.jp/PLACE/?action=newPlaceSite&site=S000028)

CARGCW8GAT 943 (-) CWWWWWWWWG [S000431](https://www.dna.affrc.go.jp/PLACE/?action=newPlaceSite&site=S000431)

CARGCW8GAT 943 (+) CWWWWWWWWG [S000431](https://www.dna.affrc.go.jp/PLACE/?action=newPlaceSite&site=S000431)

INRNTPSADB 958 (+) YTCANTYY [S000395](https://www.dna.affrc.go.jp/PLACE/?action=newPlaceSite&site=S000395)

RYREPEATLEGUMINBOX 966 (+) CATGCAY [S000100](https://www.dna.affrc.go.jp/PLACE/?action=newPlaceSite&site=S000100)

RYREPEATGMGY2 966 (+) CATGCAT [S000105](https://www.dna.affrc.go.jp/PLACE/?action=newPlaceSite&site=S000105)

RYREPEATBNNAPA 966 (+) CATGCA [S000264](https://www.dna.affrc.go.jp/PLACE/?action=newPlaceSite&site=S000264)

EBOXBNNAPA 970 (-) CANNTG [S000144](https://www.dna.affrc.go.jp/PLACE/?action=newPlaceSite&site=S000144)

MYCCONSENSUSAT 970 (-) CANNTG [S000407](https://www.dna.affrc.go.jp/PLACE/?action=newPlaceSite&site=S000407)

EBOXBNNAPA 970 (+) CANNTG [S000144](https://www.dna.affrc.go.jp/PLACE/?action=newPlaceSite&site=S000144)

MYCCONSENSUSAT 970 (+) CANNTG [S000407](https://www.dna.affrc.go.jp/PLACE/?action=newPlaceSite&site=S000407)

GTGANTG10 977 (-) GTGA [S000378](https://www.dna.affrc.go.jp/PLACE/?action=newPlaceSite&site=S000378)

RAV1AAT 980 (+) CAACA [S000314](https://www.dna.affrc.go.jp/PLACE/?action=newPlaceSite&site=S000314)

BOXLCOREDCPAL 987 (+) ACCWWCC [S000492](https://www.dna.affrc.go.jp/PLACE/?action=newPlaceSite&site=S000492)

//
