# Supplementary figures and images for: PsEND1 Is a Key Player in Pea Pollen Development Through the Modulation of Redox Homeostasis
Source: Front Plant Sci. 2021 Oct 29;12:765277. doi: 10.3389/fpls.2021.765277 (PMC8586548; doi:10.3389/fpls.2021.765277)

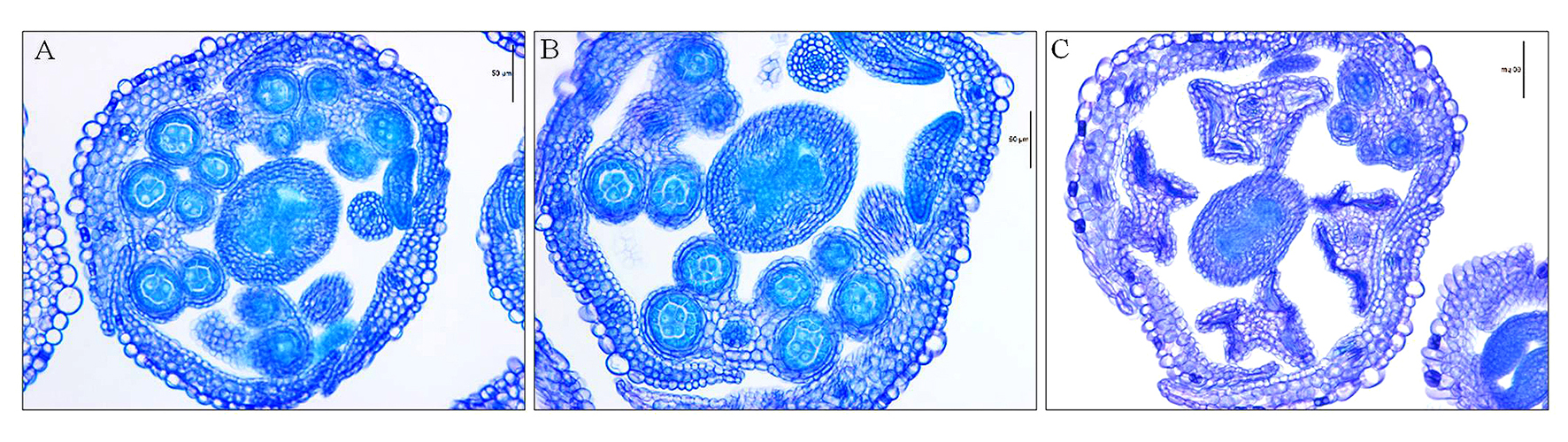

Supplement: Supplementary Figure 1 — Comparative cross-sections of WT and 35S:PsEND1 Arabidopsis thaliana flowers stained with toluidine blue. (A) Wild type; (B,C) 35S:PsEND1. At the first stages of development, we do not observe differences between anthers in (A,B), while at the stage of meiocytes (C) most of the anthers have collapsed. After this stage, the pollen mother cells and the tapetum degenerate. [file Image_1.JPEG]

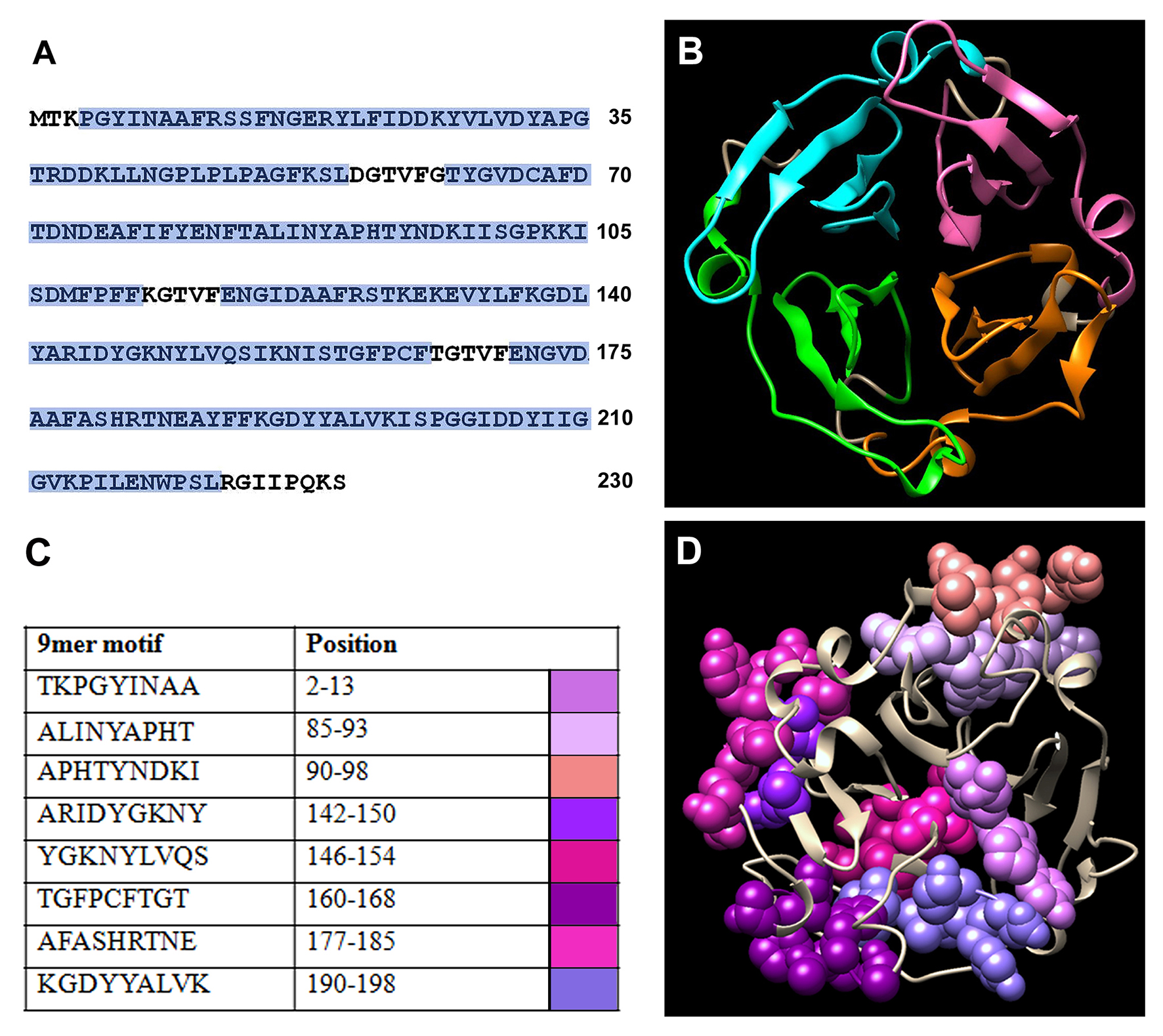

Supplement: Supplementary Figure 2 — PsEND1 sequence and predicted 3D structure showing the heme binding sites. (A) PsEND1 sequence, the hemopexin motifs are highlighted in blue boxes. (B) Predicted PsEND1 3D structure. Cyan: Hemopexin domain at position 4–55; Pink: Hemopexin domain at position 62–112; Orange: Hemopexin domain at position 118–165; Green: Hemopexin domain at position 171–222. (C) Heme binding motifs predicted by the software HeMoQuest. (D) PsEND1 3D model showing the localization of eight heme binding motifs predicted by the software HeMoQuest. The predicted heme binding sites are represented by spheres of different colors. [file Image_2.JPEG]
